# New rabies viral resources for multi-scale neural circuit mapping

**DOI:** 10.1038/s41380-024-02451-6

**Published:** 2024-02-14

**Authors:** Alexis Bouin, Ginny Wu, Orkide O. Koyuncu, Qiao Ye, Keun-Young Kim, Michele Y. Wu, Liqi Tong, Lujia Chen, Sebastien Phan, Mason R. Mackey, Ranjan Ramachandra, Mark H. Ellisman, Todd C. Holmes, Bert L. Semler, Xiangmin Xu

**Affiliations:** 1 Department of Microbiology and Molecular Genetics, School of Medicine, University of California, Irvine, CA 92697 USA; 2 Department Anatomy & Neurobiology, School of Medicine, University of California, Irvine, CA 92697 USA; 3 Department Biomedical Engineering, University of California, Irvine, CA 92697 USA; 4 The National Center for Microscopy and Imaging Research (NCMIR) and the Department of Neurosciences, School of Medicine, University of California San Diego, La Jolla, CA 92093 USA; 5 Physiology & Biophysics, School of Medicine, University of California, Irvine, CA 92697 USA; 6 The Center for Neural Circuit Mapping, University of California, Irvine, CA 92697 USA

**Keywords:** Neuroscience, Biological techniques

## Abstract

Comparisons and linkage between multiple imaging scales are essential for neural circuit connectomics. Here, we report 20 new recombinant rabies virus (RV) vectors that we have developed for multi-scale and multi-modal neural circuit mapping tools. Our new RV tools for mesoscale imaging express a range of improved fluorescent proteins. Further refinements target specific neuronal subcellular locations of interest. We demonstrate the discovery power of these new tools including the detection of detailed microstructural changes of rabies-labeled neurons in aging and Alzheimer’s disease mouse models, live imaging of neuronal activities using calcium indicators, and automated measurement of infected neurons. RVs that encode GFP and ferritin as electron microscopy (EM) and fluorescence microscopy reporters are used for dual EM and mesoscale imaging. These new viral variants significantly expand the scale and power of rabies virus-mediated neural labeling and circuit mapping across multiple imaging scales in health and disease.

## Introduction

Over the past 20 years, neurotropic viruses have been used to map neural circuits and for functional delivery of molecular markers and effectors for physiological analysis. Viral genetic tools are critical for improving anatomical mapping and functional studies of cell-type-specific and circuit-specific neural networks in the intact brain including neurological and neuropsychiatric animal models [1]. Unmet challenges are to extend existing imaging scales to finer resolution down to the electron microscopic (EM) scale and markers that allow comparison between imaging scales to help propel next generation connectomics research. To meet these goals, we developed new viral tools to expand our circuit mapping repertoire to include ultra-structural analyses combined with targeted subcellular organelle markers, and brain imaging in vivo. Our intent is to enable researchers to carry out mesoscale imaging, targeted to specific neural circuits, followed by detailed ultrastructural analyses of multiple imaging modes across scales, thus allowing direct comparison between imaging scales.

Rabies viruses (RVs) mediate efficient retrograde transsynaptic labeling in a variety of vertebrate species, including rodents and non-human primates. Genetic modifications of RVs include the addition of marker genes, adding molecular features that control synaptic spread, and pseudotyping for infection of specific selected cell types [2–9]. Wild-type rabies virus exhibits poly-synaptic spread in the brain in naturally occurring infections. To prevent its poly-synaptic spread, the gene coding for the viral glycoprotein (G protein) can be deleted so that RVΔG spread is restricted. Monosynaptic spreading is achieved by trans-complementing RVΔG with a helper virus expressing RVG protein. This system restricts recombinant RV to crossing a single synapse; providing unambiguous monosynaptic anatomical information. RVΔG can be further pseudotyped to produce virions coated with non-native envelope proteins that enable genetic targeting of specific neural circuit cell types. This system directs delivery of genetically encoded reporters and effectors to presynaptic neurons designated by their inputs [5, 6, 8, 10–13]. The Callaway group and others pioneered the development of rabies virus variants for neural circuit analysis [1, 3, 5, 8]. Osakada et al. (2011) reported a set of novel ΔG rabies variants that continue to be widely used for monitoring and manipulating neural activity, and to deliver DNA recombinases for control of gene expression. However, existing RV variants have been limited mostly to light imaging-based “mesoscale” neural circuit mapping.

We introduce here a greatly extended set of RV vectors that can be used for imaging ranges from the mesoscale to EM-level microscale. Fluorescent protein markers expressed by viral variants are very useful for cellular level imaging; we describe improved viral tools with updates based on recent progress in fluorescent protein marker brightness, stability, and photo-switch activation/molecular timer features. We show new RVΔG strains that express a range of new blue to far-red fluorescent proteins (EBFP, mTFP1, mNeonGreen, tdTomato, smURFP, and the photo-switchable “fluorescent timer”). Further improvements are conferred by expanding cell-localization-specific structural targeting; color variants can now be targeted to specific subcellular locations of interest (nucleus, plasma membrane, somatodendritic, pre- and post-synaptic specializations and mitochondria). We show a set of ferritin-based functional reporters for multi-scale neural circuit mapping.

In this study, we present proof-of-concept data attesting to the power of these new tools for discovery science. We will make these powerful new tools readily available to the neuroscience community through our established service platform at the UC Irvine Center for Neural Circuit Mapping.

## Results

During the planning stages of this work, we reasoned that a broad set of structural and functional reporters would be valuable for extending RV applications for the broad neuroscience community. We designed, produced, and tested our novel RV reagents to determine their utility for multi-channel or multi-modal imaging of multiple reporters and labels in the experimental preparations. Using the SAD-B19-RVΔG derived from an attenuated vaccine virus as a starting point, we developed modifications, creating a new series of recombinant RVs that encode new fluorescent markers and novel functional reporters (Table 1). We generated a series of RVΔGs that express a range of reporters for multi-color imaging, including spectrally distinguishable blue (EBFP), turquoise (mTurquoise2), teal (mTFP1), green (mNeonGreen), red (tdTomato), and far-red (smURFP) proteins that can be targeted to specific subcellular locations. We further extended this to develop RV variants that express ferritin and luciferase to allow for micro- (EM) to meso- (fluorescent labeling) level circuit mapping.

**Table 1:** Summary of new recombinant rabies virus variants.

Name	Reporter	Localization	Excitation (nm)	Emission (nm)	Relative Brightness^a^	Insert length (nt)
SAD-B19-RVΔG EBFP	EBFP	Cytoplasm	380	440	6.3	720
SAD-B19-RVΔG mTurquoise2	mTurquoise2	Cytoplasm	434	474	27.9	720
SAD-B19-RVΔG mTFP1	mTFP1	Cytoplasm	462	492	54.4	711
SAD-B19-RVΔG mNeonGreen	mNeonGreen	Cytoplasm	506	517	92.8	711
SAD-B19-RVΔG tdTomato	tdTomato	Cytoplasm	554	581	95.22	1431
SAD-B19-RVΔG smURFP	smURFP	Cytoplasm	642	670	32.4	402
SAD-B19-RVΔG Slow-FT (blue/red)	Slow fluorescent timer (FT)^b^ (modified mCherry)	Cytoplasm	402/583	465/604	11.7/4.2	711
SAD-B19-RVΔG PSD95-mNeonGreen	mNeonGreen	Postsynaptic (via PSD95)	506	517	92.8	2937
SAD-B19-RVΔG SNPN-tdTomato	tdTomato	Presynaptic (via synaptophysin)	554	581	95.22	2367
SAD-B19-RVΔG ChR2-EYFP-Kv	EYFP	Somatodendritic	513	527	44.89	1860
SAD-B19-RVΔG 1XNLS-tdTomato	tdTomato	Nucleus (via 1x nucleus localization signal)	554	581	95.22	1503
SAD-B19-RVΔG 2XNLS-tdTomato	tdTomato	Nucleus (via 2x nucleus localization signal)	554	581	95.22	1503
SAD-B19-RVΔG MLS-tdTomato	tdTomato	Membrane (via membrane localization signal)	554	581	95.22	1476
SAD-B19-RVΔG 2XMito-dTomato	dTomato	Mitochondria (via 2x cytochrome C localization)	554	581	47.61	702
SAD-B19-RVΔG mNeonGreen-T2A-2XMito-dTomato	mNeonGreen	Cytoplasm	506	517	92.8	711
	dTomato	Mitochondria (via 2x cytochrome C localization)	554	581	47.61	702
SAD-B19-RVΔG tdTomato-T2A-Nluc	NanoLuc luciferase (NLuc) & tdTomato	Cytoplasm	554	581	95.22	1431
SAD-B19-RVΔG EGFP-FTL	Ferritin & EGFP	Cytoplasm	488	507	33.54	717
SAD-B19-RVΔG emGFP-T2A-FTL	Ferritin & emGFP	Cytoplasm	487	509	39.1	717
SAD-B19-RVΔG emGFP-T2A-MLS-FTL	Ferritin & emGFP	Membrane	487	509	39.1	717
SAD-B19-RVΔG GCaMP7f	GCaMP7f	Cytoplasm	480	510	45.0	1353

^a^Note that fluorescent molecular brightness is calculated as the product of extinction coefficient and quantum yield.

^b^The fluorescent timer (FT) is derived from a directed molecular evolution of mCherry, and the FT exhibits protein-maturation-time-dependent fluorescence changes between the protonated blue GFP-like and the anionic red DsRed-like chromophore states.

### Construction of recombinant rabies viruses

**Fig. 1:**
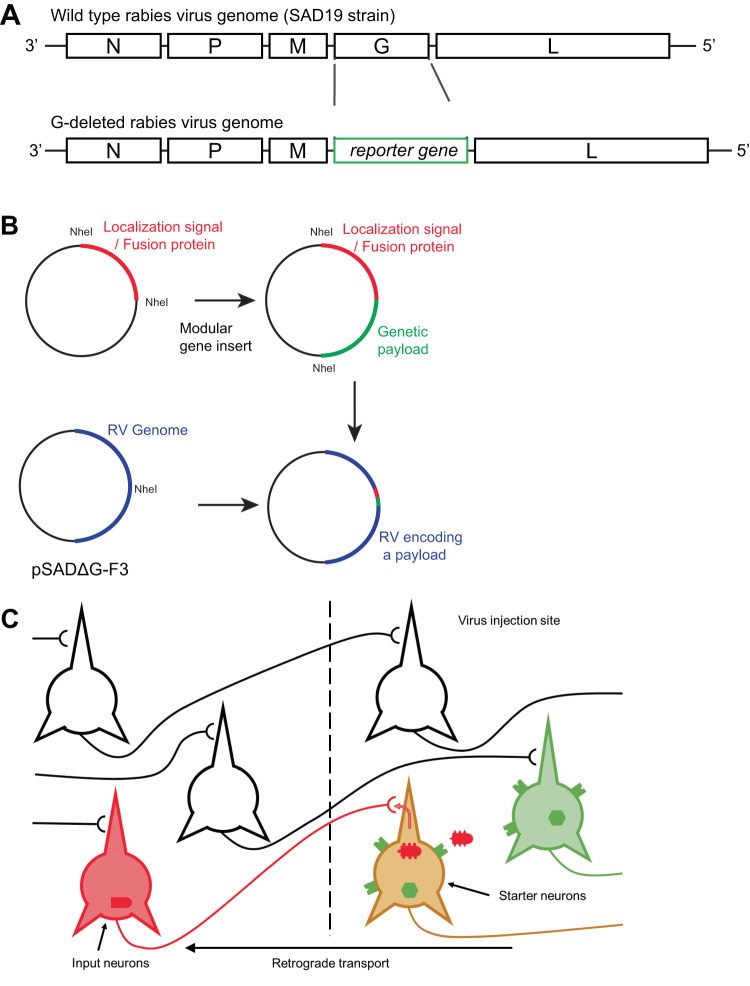
Cloning of recombinant rabies virus for monosynaptic neural tracing. **A** Schematic representation of WT rabies virus (RV) genomic RNA (top) and the modified recombinant fluorescent rabies virus genomic RNA (bottom). **B** Cloning strategy. Reporter genes were cloned in a vector downstream a localization signal under the control of a CMV promoter. Reporter properties were assessed after transfection of HeLa cells. Reporter genes were then cloned in the RV cDNA in place of G gene. **C** Schematic representation of monosynaptically restricted RV spread. EnvA-pseudotyped RV (red) and helper AAV (green) co-infect starter neurons. Helper AAV expression provides the TVA receptor for starter neuron infection and glycoprotein for trans-synaptic spread. Starter cells produce both red and green fluorescent signals. Presynaptic input neurons forming synapses with starter neurons are infected and produce red signals allowing for specific identification of the neural circuit connections.

We extended earlier technology to use modular gene inserts that allow efficient generation of recombinant rabies viruses. To generate recombinant RVΔG, we started with plasmids encoding a cDNA of the SAD-B19 rabies virus strain genome harboring two transcription start and stop signals in place of the G (viral glycoprotein) gene [8, 14]. This template allowed the easy insertion of a gene of interest in place of the glycoprotein gene (Fig. 1A) by restriction enzyme cloning and modular expression of different reporters or payloads. Prior to generating a recombinant RVΔG expressing a heterologous gene, we analyzed the expression of the heterologous gene using a mammalian cell expression vector under the control of the early CMV promoter to provide us with an initial verification of expected properties of the protein (e.g., fluorescence, cellular localization). As CMV promoter-mediated expression of reporters is more efficient than viral expression, it allows for detection of leaky expression more efficiently in cell culture. Reporter genes were then transferred to plasmids harboring the complete RV genome (Fig. 1B). Final recombinant plasmids of RVΔG were verified by recombinant reporter gene sequencing.

Plasmids expressing the cDNA sequence of SAD-B19-RVΔG were co-transfected in cultured B7GG cells with plasmids encoding the viral nucleoprotein (N), phosphoprotein (P), large protein (L) and glycoprotein (G). B7GG cells stably express T7 polymerase, a nucleus-localized GFP, and the viral G protein. These features allow for the replication, egress, and reentry of the virus in culture, providing a safe and efficient way to amplify the virus to high-level titers. While the RVΔG virus has its envelope glycoprotein gene deleted from the genome, it is grown in complementing B7GG cells so that the glycoprotein is incorporated into its viral particles. Thus the RVΔG virus can infect permissive cells.

Amplified stocks of viral vectors were propagated and used in our subsequent cell culture and animal injection experiments. We use un-pseudotyped RVΔG to infect BHK-EnvA cells; the production of the EnvA-pseudotyped RVΔG follows an earlier published protocol [13]. Viral titers of ~5 ×10^8^ infectious units /ml for un-pseudotyped and EnvA-pseudotyped RVΔG are in the effective application range.

Pseudotyped RVΔG viruses are designed to be used as monosynaptic tracers in combination with the helper adeno-associated virus (AAV) expressing RV glycoprotein. Briefly, helper AAVs expressing TVA (EnvA receptor) and rabies glycoprotein genes are injected in mouse brain to infect neurons. Fourteen to twenty-one days after AAV infection, EnvA-pseudotyped rabies virus is injected in the same target region, allowing for the specific infections of neurons expressing TVA (Fig. 1C). The glycoprotein expression defect of recombinant RVs is rescued by helper AAV protein expression, resulting in a monosynaptic spreading that allows the identification of input neurons from the initial injection site.

### Recombinant RV clones expressing cytoplasmic reporters

**Fig. 2:**
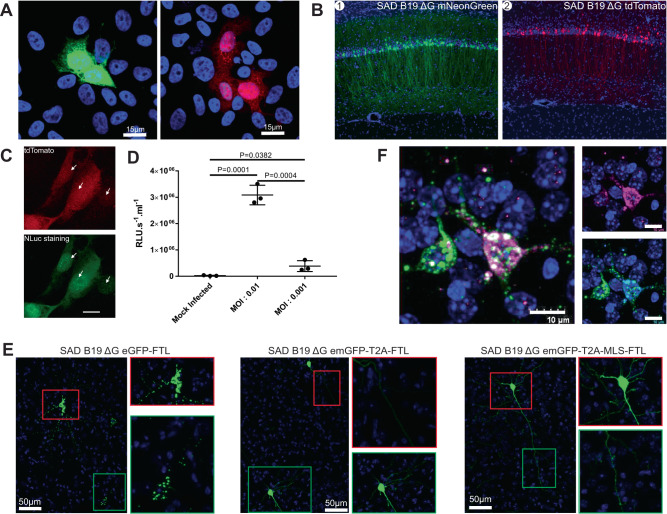
Recombinant rabies virus express cytoplasmic reporters. **A** Vero cells were infected with recombinant rabies virus expressing mNeonGreen (1) and tdTomato (2). At 48hpi cells were then fixed in 3.7% formaldehyde, DAPI staining was performed, and slides were mounted and imaged. **B** Retrograde labeling of hippocampal CA1 pyramidal cells, following injection of different RVΔG viruses in mouse dorsal subiculum: RVΔG mNeonGreen shown in green (**B1**) RVΔG tdTomato shown in red (**B2**). **C** Vero cells were infected with rabies virus expressing tdTomato and nanoluciferase. At 72hpi cells were fixed and immunofluorescent assay targeting nanoluciferase was performed. Cells were imaged for tdTomato (red fluorescent emission, 1) and nanoluciferase (immunocytochemistry, green label, 2, white arrows). **D** Multiplicity of infection of 0.01 and 0.001 were used to infect B7GG cells. At 48 hpi cells were harvested and luminescence was quantified using a luminometer, showing that viral infection titer and luminescence signal scales, thus expression levels can be controlled by infection titer. Statistical analysis was performed using an unpaired t-test in Graphpad prism. **E** Characteristic differences in labeling between RVΔG eGFP-FTL (ferritin fused with eGFP), RVΔG emGFP-T2A-FTL (emGFP cleaved from ferritin), and RVΔG emGFP-MLS-FTL (membrane targeted emGFP) in neuronal cell bodies and dendritic projections. **F** Neurons infected with RVΔG eGFP-FTL and immunofluorescent stained with anti-ferritin light Chain antibody followed by Cy5 AffiniPure Donkey Anti-Rabbit IgG (H + L). The blue signal is for DAPI staining (nuclei), the green signal is for the green fluorescent protein (EGFP), and magenta staining results from immunofluorescent labeling of ferritin light chain.

We generated RVΔG recombinants carrying the genes for new fluorescent proteins. We developed several viral constructs expressing cytoplasmic reporters (Fig. 2, Fig. S1A–F). The brightest fluorescent reporters are mNeonGreen and tdTomato (Figs. 2A, B, S1D, E). For a specific green signal, our new RVΔG variant expressing mNeonGreen (Fig. 2A1 and 2B1) is a particularly good choice. The mNeonGreen signal is very bright, stable and is excluded with DAPI and EBFP filter sets. Our brightest reporter RVΔG variant expresses tdTomato (Fig. 2A2 and 2B2). The tdTomato signal is strong and stable. This red signal is best detected using widely available Texas Red filter sets. The signal is readily observed in both cell culture (Fig. 2A) and in brain slices of infected mice (Fig. 2B). These reporter fluorescent signals are brighter than the usual eGFP or mCherry signals used in other reporters. Infected cultured cells displayed a detectable signal at 24 h post infection (hpi) in Vero cells and produce a strong signal at 48 hpi (Fig. 2A). Diffusible fluorescent reporters fill the entire cytoplasm in cultured cells without aggregation on specific organelles. Images displayed in Fig. 2B resulted from an incubation of 7–8 days in C57BL/6 background mice. Cell bodies appear very bright, and subcellular signals in the axons and dendrites are observed.

Additional constructs expressing cytoplasmic reporters were produced. Below, we describe each one in order of peak wavelength sensitivity. Our first recombinant RVΔG expresses EBFP (Fig. S1A), a blue fluorescent protein. The EBFP signal is stable but faint, thus counterstaining with DAPI (or any other blue dye) is not recommended. We also generated RVΔG variants that express mTurquoise2 and monomeric teal fluorescent protein (TFP1) (Figs. S1B and S1C). These two new fluorescent proteins produce bright signals that are detectable with standard DAPI and FITC filter sets. For co-staining applications using these recombinant viruses, the use of filters with narrower ranges is recommended to prevent signal bleed-through between blue and green channels. To extend the spectral range into far-red, we developed a RVΔG variant that expresses smURFP (Fig. S1F). The smURFP signal is readily detectable but requires the addition of biliverdin (20 µM) 24 h before imaging. The smURFP-biliverdin complex has a half life of 33–35 h [15]. Far-red reporters are not as common as the other reporters described in this study, but they confer multiple experimental advantages that include increased imaging depth, lower phototoxicity, and a reduction in auto-fluorescent background noise that commonly interferes with using green and red fluorescent reporters [16].

After engineering recombinant rabies viruses that express reporters with a wide spectral range of different wavelengths and different sub-cellular localization, we constructed an RV variant that expresses a fluorescent timer (FT) protein [17] within the RVΔG genome. The FTs are derived from mCherry and initially produce a blue signal. Over the multi-hour course of its maturation, the fluorescent signal gradually changes from blue to red. At 37°C, Slow-FT blue fluorescence peaks at 9.8 h and, over the course of the subsequent 18 h, switches to red. Based on the signal observed, this recombinant virus is useful for analyzing the timing of payload delivery from the same backbone RV modular construct. Newly infected cells appear blue, while red cells are characteristic of infections that last for more than 30 h. While we used Slow-FT as a model for this study, other timer variants exist. Fast and medium-FT exhibit faster rates of maturation than Slow-FT. As seen in Fig. S1M, all infected cells appear blue due to the constant synthesis of Slow-FT. The newly infected cells only display a faint blue signal (white arrow), while cells sustaining a longer infection also appear red. Similar timer proteins modified to exhibit different maturation times can be expressed by recombinant viruses to fit more specific needs of the user.

In addition to these cytoplasmic fluorescent reporters, we developed a recombinant virus expressing both tdTomato and nanoluciferase (NLuc). We constructed RVΔG vectors expressing NLuc (Fig. 2C, D), a small (171 amino acids) engineered luminescent protein that converts furimazine to furimamide, thus producing bright luminescence [18, 19]. This nanoluciferase reporter is linked to tdTomato along with a “self-cleavable” T2A sequence [20] inserted between the two coding sequences. Luciferase applications can extend to the level of in vivo whole animal imaging and are critical for biological contexts that preclude the use of exogenous light excitation or for light sensitive physiological processes [21, 22]. The synthesis of tdTomato, assessed by live imaging (Fig. 2C), shows a strong red signal 48 h post infection (hpi) of B7GG cells. NLuc synthesis can be detected by immunofluorescent staining of infected B7GG cells (Fig. 2C) and exhibits expression timing similar to that of tdTomato. A dose-dependence analysis was performed by infecting B7GG cells using increasing amounts of virus (Fig. 2D). The luminescent signal observed was quantified at 48 hpi on a luminometer and correlates with the viral solution volume used for infection with an average RLU/sec/ml value of 15,432 and 1924 for infection with MOI : 0.01 and 0.001, respectively. NLuc-based luminescence imaging can be used to quantify viral protein synthesis in cell or tissue extracts, and it can also be used for live imaging in small animals. Unlike fluorescence imaging, which is readily detectable, live imaging of nanoluciferase requires specialized imaging systems and substrate [21, 22]; however, it obviates the need for exogenous light excitation and the attendant potential issues of phototoxicity or light activation of endogenous light sensitive elements.

Fluorescent reporters have been used extensively for neural circuit mapping, but their utility is limited by the spectral wavelength ranges, excitation wavelength depth penetration and by the spatial resolution of fluorescence microscopy technologies. To expand the range of detection with recombinant RVs, we have developed new functional reporters and coupled them with fluorescent proteins. To expand the imaging scale of rabies tools, we constructed dual-utility RVΔG vectors to extend the multi-scale imaging ranges to EM imaging by rabies variants that genetically encode enhanced ferritin. Ferritin light chain binds iron and forms a multimeric sphere containing 24 subunits of ferritin surrounding a core of hydrous ferric oxide [23]. These ferric cores are readily detectable using immunostaining, electron microscopy and MRI techniques. We designed recombinant RV encoding ferritin light chain from which three variants were engineered: a fusion between EGFP and ferritin light chain (RVΔG EGFP-FTL), a reporter harboring a T2A sequence between emerald GFP (emGFP) and ferritin light chain (RVΔG emGFP-T2A-FTL), and a third variant with a T2A sequence separating emGFP and ferritin light chain fused to a membrane localization signal (RVΔG emGFP-T2A-MLS-FTL). emGFP was used in the later variants as it has improved photostability and brightness compared to EGFP, as well as its superior folding at 37 °C [24]. Expression of ferritin in cultured Vero cells (Supplementary Fig. S2) and in murine model (Fig. 2F) was assessed by immunostaining. Localization in neurons following intracranial injection was monitored after 7–8 days. The fused version of ferritin with eGFP lead to the visualization of puncta in the cell body and processes, and versions harboring cleaved version of the reporters produce a strong fluorescent cytoplasmic signal (Fig. 2E).

### Generation of recombinant rabies virus expressing reporters for studying specific sub-cellular compartments

**Fig. 3:**
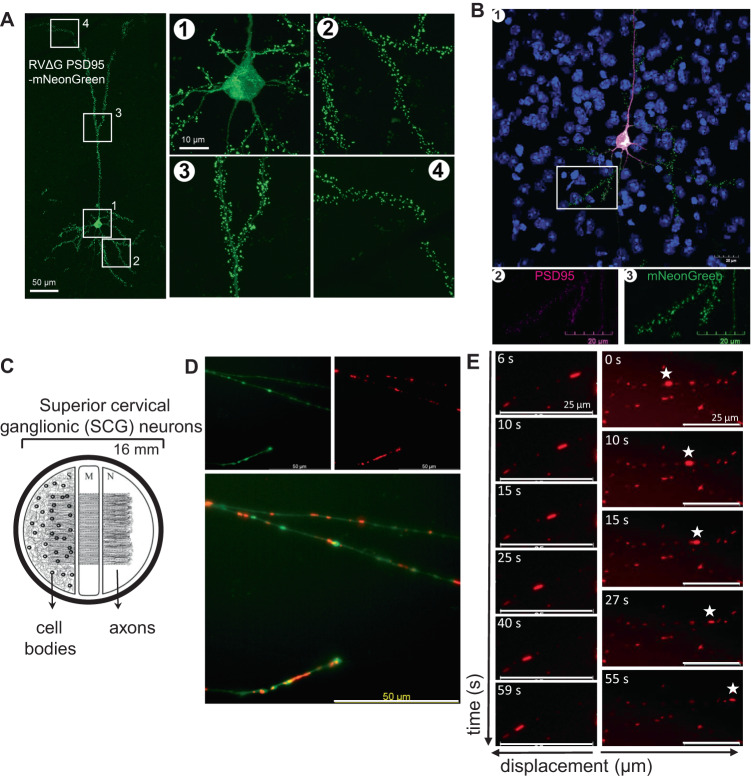
Recombinant rabies viruses label specific sub-cellular compartments with targeted fluorescent reporters. **A** RVΔG PSD95-mNeonGreen (green) labeling of a cortical pyramidal cell. Left panel shows a 20x confocal image of the labeled cell. A1-A4 show 60x confocal images with cellular details showing the soma, axon, and basal dendrites with clearly visible dendritic spines (1), basal dendritic spines (2), dendritic spines of the apical trunk and dendritic branching (3), and dendritic spines at the apical tuft (distal apical dendrites) (4). **B** Immunofluorescent staining of PSD95 reveals a near perfect colocalization of virus-expressed PSD95 (green fluorescent signal) with total cell PSD95 (antibody targeted against PSD95, magenta fluorescent signal). **C** Schematic of a tri-chamber (S: soma, M: middle/methocel, N: neurite). **D** Neurons were infected with RVΔG mNeonGreen-T2A-2Xmito-dTomato, axons were imaged starting at 24 hpi. Green fluorescent signal produced by mNeonGreen can be observed in the entire axon, and red punctae are produced by dTomato localized on mitochondria, allowing for tracing of mitochondria transport along the axon (see Supplementary Movie 1). Movie was acquired from the S compartment close to the M compartment. **E** For single mitochondria tracking in axons, neurons were infected with RVΔG 2Xmito-dTomato and continuous movies were recorded (app. three frames/s) for 1 min, and representative frames are shown (scale bar is 25 µm, please see Supplementary Movie 2). Movie was acquired from the S compartment close to the M compartment.

To extend the recombinant RV repertoire and provide tools to study specific sub-cellular compartments, we refined the spatial distribution of fluorescent reporter signals. We generated RVΔG variants that encode reporters targeted to specific sub-cellular locations by fusion with localization protein sequences (Fig. 3A, B and Fig. S1H–K) and organelle-specific intracellular compartments (Figs. 3D, E, S1G and S1L). Data presented in Fig. 3A, B were imaged 8 days after injection of RVΔG variants expressing a fusion protein of PSD95 and mNeonGreen in C57BL/6 background mice. Infected neurons produced strong green fluorescent signals in cell bodies and puncta in dendrites. Since viral replication and protein synthesis occurs in the cell body, we expected to detect fluorescent signal on replication organelles in the cytoplasm. The fluorescent fusion proteins subsequently migrate to dendritic spines and produce characteristic labels of dendritic spines (Fig. 3A). To validate our viral labels, immunostaining of PSD95 was performed. Almost a perfect colocalization of green signals produced by mNeonGreen and far-red signals resulting from the immunofluorescent staining is observed (Fig. 3B). While images show a very good correlation of localization, automated counting was much more efficient at detecting RV-mediated fluorescent protein signal with 128 spines detected compared to antibody-based detection, which is slightly less efficient for detection, yielding 100 spines. Overall, the fluorescent protein signal versus antibody-based signal measurements yield qualitatively similar results in spite of these differences in signal detection efficiency. mNeonGreen produces a much brighter signal without background noise while antibody staining produces a fainter signal, which makes it more difficult to detect using available software. Since B7GG cells do not form synapses in culture, it is not possible to evaluate the specificity of the subcellular labeling in cell culture (Fig. S1J); however, infection of B7GG cells with this RVΔG PSD95-mNeonGreen recombinant virus results in a bright fluorescent cytoplasmic signal.

We generated RV vectors with targeted organelle-specific reporter expression. We engineered a recombinant virus expressing dTomato flanked by two copies of the mitochondria localization signal derived from cytochrome C [25], with or without co-expression of cytoplasmic mNeonGreen. When the reporter localizes to mitochondria, the close proximity of different dTomato molecules facilitates dimerization and produces strong red fluorescent signals within these organelles (Fig. S1L). Mitochondria are essential for metabolic processes including ATP production, calcium homeostasis, and apoptosis. In neurons, the spatial location of mitochondria is highly dynamic, and mitochondria are located as needed by cellular compartments with increased energy demands such as axonal growth cones and synapses [26, 27]. Disrupted mitochondrial function and dynamics are associated with neurodegenerative diseases, neuropathies [28], and microbial infections [29, 30]. Mitochondria-selective dyes (e.g., MitoTracker-red) have made it possible to track mitochondria in living cells [29]. However, such cell-permeable dyes are restricted to short-term imaging experiments and have additional issues of high background and photobleaching. These experimental issues, particularly in neuronal tissues, limit their applications and utility for long term longitudinal analyses. Recombinant rabies viruses can be targeted to specific cell types based on cellular identity or connectivity, and our new RV reporters allow imaging and characterization of mitochondrial dynamics in axons with high precision and minimal background. To test the new RV reporters in neuronal culture and to monitor the fluorescent marker expression using time-lapse microscopy analyses, we used a specialized culture system that effectively divides somatodendritic and axonal compartments in tri-chambers (Fig. 3C). RVΔG mNeonGreen-T2A-2XMito-dTomato infection of SCGs produces a strong cytoplasmic green, fluorescent signal coupled with strong red fluorescent signal localized to mitochondria (Fig. 3D). Live-cell imaging allowed the detection of mitochondria traveling along the axons of infected SCG neurons (Supplementary Movies 1, 2). RVΔG 2XMito-dTomato infection of SCG neurons shows very strong cell body fluorescence at 24 hpi (Fig. 3E). Abundant mitochondria localization in axons is detected at this time point. Axonal mitochondria have a range of sizes and shapes (Fig. 3E), and a subset are motile. We recorded real-time motility of single/multiple mitochondria (Supplementary Movies 1, 2), and extracted representative frames to demonstrate different dynamics of axonal mitochondria (Fig. 3E). We quantified the speed of motile mitochondria presented in Fig. 3D, E, which were 2 and 2.7 µm/s respectively.

Additionally, we engineered RVΔG variants that encode tdTomato flanked by a SV40 nuclear localization signal (NLS) (1x at the N terminus and 2x at both the N and C terminus). We find strong nuclear localization of tdTomato using such variants (Fig. S1G). To target cell membranes, we constructed a version of RVΔG expressing tdTomato fused to the neuromodulin-associated plasma membrane localization sequence (MLS) [31]. Although this recombinant virus resulted in some non-specific labeling in B7GG cells (Fig. S1H), there is relatively homogeneous labeling of plasma membranes of the cells, with no nuclear accumulation. We designed other RVs to target signals to somatodendritic locations (RVΔG ChR2-EYFP-Kv, Fig. S1I) and presynaptic structures (RVΔG SNPN-tdTomato, Fig. S1K, Fig. S3), respectively, through the use of Kv2.1 targeting motif [32, 33] and synaptophysin fusion.

**Fig. 4:**
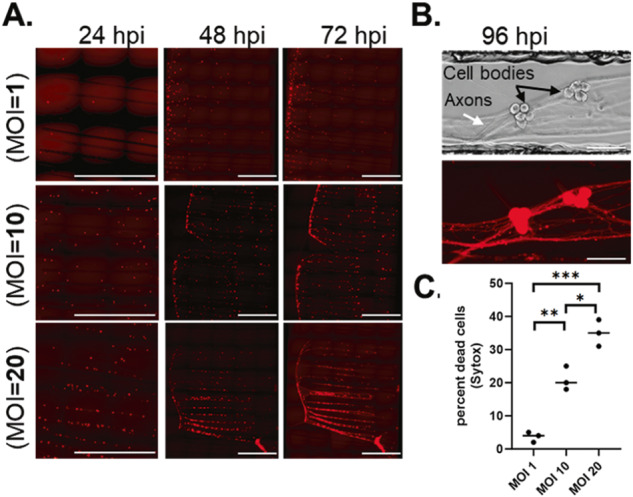
RV fluorescent protein expression kinetics and toxicity at different multiplicities of infection (MOI) in compartmented primary neuronal cultures. **A** Primary rat neurons (SGCs) were seeded in the S-compartments of tri-chambers. On day 0, cells were infected with RVΔG dTomato (dT) using MOI: 1, 10, and 20. Whole S-compartments were scanned at 24, 48, 72 h post infection (hpi) (scale bar is 2 mm). **B** Higher magnification images of neuron cell body clusters (black arrows) and axon bundles (white arrow) showing diffuse dT expression at 96 hpi (1 MOI infection) (scale bar is 100 µm). **C** At 96 hpi, neurotoxicity was quantified using Sytox stain. Ratio of dT and GFP positive (dead) neurons over dT positive neurons are shown in the graph as percentage. Statistical analysis was performed using an unpaired *t*-test in Graphpad prism. * *p* = 0.01; ** *p* = 0.0016; *** *p* = 0.0002.

To determine the infection dynamics of the novel RVΔG recombinants at different multiplicities of infection (MOI), we infected primary superior ganglionic neurons that were cultured in compartmented chambers. We infected S-compartments with RVΔG-tdTomato at an MOI of 1, 10, or 20, and monitored the fluorophore expression kinetics and neurotoxicity over time (Fig. 4). Almost all of the neurons produce strong red fluorescent signals at 24 hpi when infected at an MOI of 10 or 20, while less than 1% of neurons show detectable tdTomato expression at an MOI of 1 at this time point. At 48 and 72 hpi, infection at an MOI of 1 show expression comparable to neurons infected at an MOI of 10 or 20. Furthermore, we detected that the toxicity of the RVΔG is significantly less (<5%) during an infection at an MOI of 1 at 96 hpi, compared to infections at the higher MOI’s (quantified in Fig. 4C). Fluorophore expression is detected in cell bodies as well as proximal and distal axons at this time point (Fig. 4B). In conclusion, these data show that RVΔG infections at high MOI result in faster expression of transgenes at a cost of neurotoxicity, since these vectors are spread-deficient but replication competent. For long term or functional analyses, low MOI infections are preferrable with delayed expression kinetics but reduced toxicity.

**Fig. 5:**
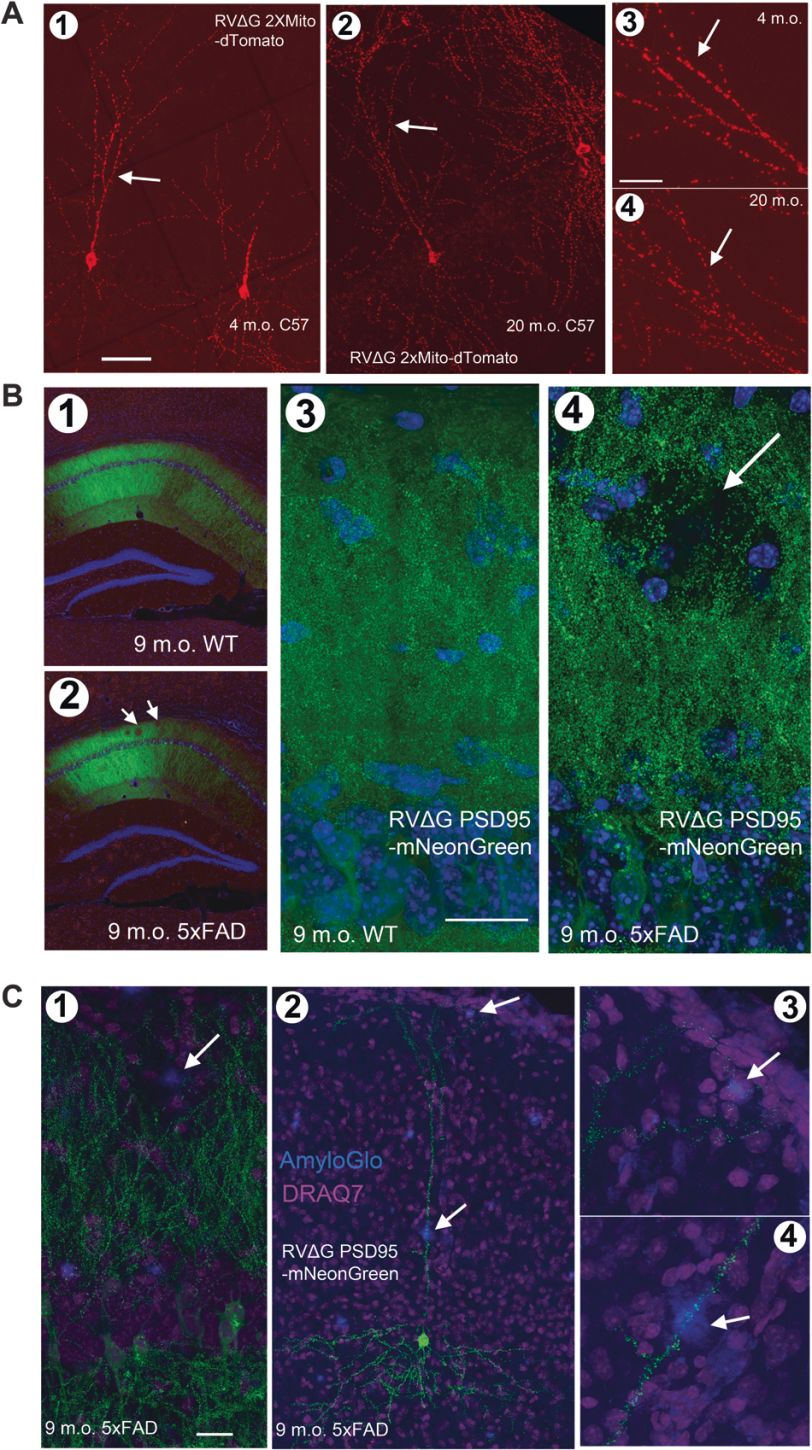
Recombinant rabies virus reveals synaptic structural changes in Alzheimer disease model mice. **A** RVΔG 2Xmito-tdTomato (red) labeling of mitochondria in hippocampal CA1 pyramidal cells in a 4-month-old C57 mouse (1), and in a 20-month-old C57 mouse (2). **3**–**4** show 60x confocal images with cellular details showing distinguishable features of labeled mitochondria in the apical dendrites of the excitatory neurons in mice at different ages. **B** RVΔG PSD95-mNeonGreen (green) labeling of hippocampal CA1 in 9-month-old C57 wild type (1) and 5xFAD (2) mice, counterstained with DAPI (blue). Dense dendritic spines are seen in area CA1 of both mice, but the stratum oriens layer of the 5xFAD mouse contains noticeable regions with reduction of dendritic spine densities that are indicated with arrows (**2**, **4**). 5xFAD CA1 shows significant dendritic and synaptic losses at the age of 9 months compared to the wild type mouse. **C** RVΔG PSD95-mNeonGreen (green) labeling of hippocampal and cortical pyramidal neurons in the 9-month-old 5xFAD mouse. AmyloGlo staining (blue) reveals beta-amyloid plaques, DRAQ7 (violet) counterstains the nuclei. Hippocampal CA1 cells show localized dendritic spine loss within amyloid plaques as indicated by AmyloGlo staining (1). Sparse labeling of a cortical pyramidal cell shows locally reduced dendritic spines adjacent to amyloid plaques (**2**–**4**).

To showcase the research discovery potential of these new tools, we used RVΔG 2XMito-dTomato to measure microstructural changes of labeled neurons in aging and Alzheimer’s disease mouse models. Young (4 month old) and old (20-month old) mice were injected with RVΔG 2XMito-dTomato in the subiculum. As CA1 excitatory cells project to the subiculum, the hippocampal CA1 pyramidal cells are retrogradely labeled with RVΔG 2XMito-dTomato (Fig. 5A). Using this new recombinant RVΔG, we can effectively perform sensitive measurements of dTomato-expressing mitochondria; on average, mitochondria are 20% smaller in old hippocampal CA1 pyramidal cells than in young hippocampal CA1 pyramidal cells (0.70 ± 0.03 μm^2^ in young CA1 cells vs. 0.56 ± 0.02 μm^2^ in old CA1 cells, mean ± SEM, *p* < 0.0001, LME). These data demonstrate the utility of RVΔG-2XMito-dTomato in characterizing mitochondrial morphological changes linked to aging. Use of these novel RV recombinants allows characterization of neuronal micro-structures and subcellular organelles in healthy and disease model neurons.

As a further proof of concept, we used RVΔG PSD95-mNeonGreen to examine structural synaptic loss of hippocampal and cortical excitatory neurons in 5xFAD Alzheimer’s disease model mice. At 9 months of age, 5xFAD mice have amyloid-β plaques in hippocampal CA1 (arrows, Fig. 5B, 1–4), compared to 9 month old age-matched wild type C57BL/6 mice. We identify amyloid-β plaques using AmyloGlo (a chemical marker for amyloid) and counterstained cell nuclei with DRAQ7, a nuclear stain. We observe “fuzzy” amyloid plaques in the empty space where dendritic spines and synapses are expected to be found in wild type mice but are absent in 5xFAD mice (Fig. 5C1). This is in line with previous reports of an age-related increase in synaptic loss in 5xFAD mice [34, 35]. In addition to studying densely labeled neurons, we examined sparse labeling of neuronal elements in close proximity to amyloid-β plaques (Fig. 5C, 2–4). Proximal or distal apical dendrites passing through or around the amyloid beta plaques show decreases in the dendritic spine density and intensity, supporting our hypothesis that amyloid-β plaques may damage the dendrite structures and alter previously existing synaptic connections. We analyzed the effect of beta-amyloid plaques detected with AmyloGlo on the density of dendritic spines labeled by RVΔG PSD95-mNeonGreen shown in Fig. 5C, 3–4. We compared three segments of the dendrites ranging from the closest to the furthest to the center of beta-amyloid plaques. A significant decrease in the number of dendritic spines on dendrite segments closest to the beta-amyloid plaque was observed (normalized spine number value: 0.177 ± 0.014 (mean ± SD)) compared to two dendrite segments further away from the beta-amyloid plaque (middle segments: 0.365 ± 0.039; distal segments: 0.458 ± 0.042) (measurements from three samples; *p* = 0.0107 Kruskal-Wallis test).

### In vivo functional imaging of neuronal activity using RVΔG expressing GCaMP7f

**Fig. 6:**
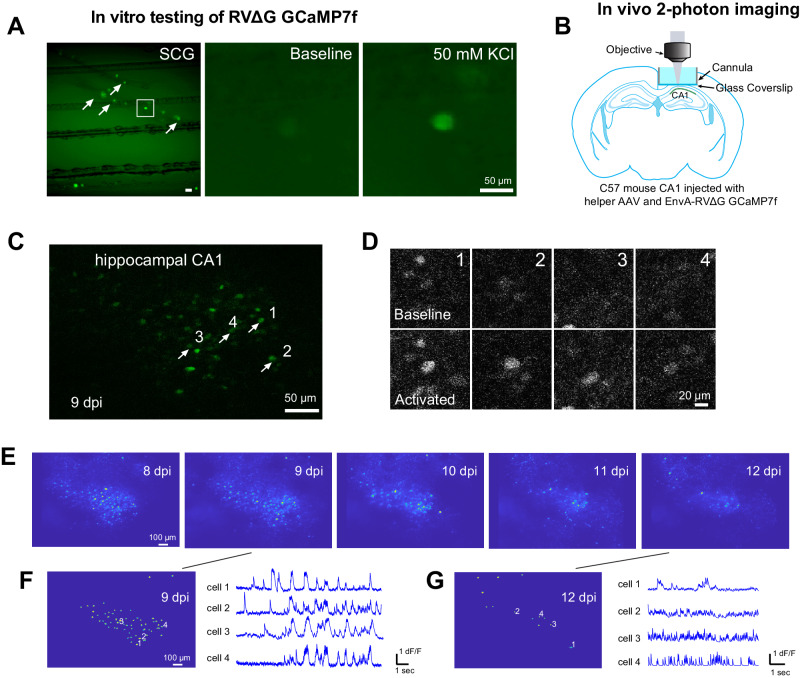
In vivo functional imaging of neuronal activity using RVΔG expressing GCaMP7f. **A** SCG primary rat neurons were infected with unpseudotyped SAD-RVΔG GCaMP7f and treated with 50 µM KCl to depolarize the neurons. Increased green fluorescent protein brightness is seen in response to KCl induced depolarization. The left panel shows cultured SCG cells after KCl-induced calcium influx. The middle and right panels show a representative cell magnified from the left panel, before and after 50 mM KCl-induced calcium influx. **B** Schematic illustration of in vivo two-photon calcium imaging of mouse hippocampal CA1. The helper AAV and EnvA-RVΔG GCaMP7f viruses were injected into the hippocampal CA1 region of C57 mice. **C** Top-down view of mouse hippocampal CA1 excitatory neurons by 2-photon imaging at day 9 post RV injection (9 dpi). **D** Examples of activated neurons with RVΔG GCaMP7f-mediated 2-photon calcium imaging from (**C**). **E**–**G** Longitudinal imaging (8–12 dpi) of RVΔG GCaMP7f infected CA1 neurons in the same field of view shows an appropriate time window (8–10 dpi) for the RVΔG GCaMP7f-mediated imaging application. The maximum projection images of GCaMP7f fluorescence are shown for each day in (**E**), while the CNMFE-extracted functional active neurons are shown for different time points in (**F**) (8 dpi) and (**G**) (12 dpi) with example calcium signal traces on the right.

We engineered a RVΔG recombinant virus expressing GCaMP7f for in vivo monitoring of neural activities. GCaMP-based reporters are calcium indicators based on a fusion of cpGFP, calmodulin, and M13 [36]. Increased brightness of the fluorescent signal is observed upon calcium binding to the GCaMP reporter, thus allowing rapid detection of neuronal calcium dynamics in vivo. RVΔG GCaMP7f was initially tested in cell culture. Figure 6A presents the example result from live imaging of primary SCGs infected with RVΔG GCaMP7f. To activate SCG cells, we added 50 mM KCl to the culture medium. We observe GCaMP7f-mediated responses within seconds of the KCl application, shown by a robust increase of green fluorescent signals in infected cell bodies (Fig. 6A). In vivo experiments were then performed in C57/BL6 mice, which were injected with helper AAV (AAV8-DIO-TC66T-2A-mCherry-2A-oG and AAV1-Camk-cre) and EnvA-pseudotyped RVΔG GCaMP7f in hippocampal CA1 to label excitatory neurons and their directly connected neural networks. The helper AAV was injected on day 1, followed by rabies virus injection 21 days later. Immediately after the rabies injection, we performed in vivo two-photon imaging of the exposed hippocampal surface (Fig. 6B). Two-photon live calcium imaging allows us to map large CA1 network activities in single-cell resolution in vivo (Fig. 6C–E). Calcium imaging was recorded in awake mice, and individual spontaneous neuronal calcium responses were monitored and analyzed (Fig. 6C, D; Supplementary Movie 3). GCaMP7f-expressing CA1 cells are detectable by day 4–5 post RV injection (4–5 dpi) with 2-photon microscopy, and healthy single-cell calcium activities are observed during 7–10 dpi with spontaneously active neurons displaying clearly detectable increases in fluorescence signals compared to resting baseline fluorescence (Fig. 6E, F). Note that decreased numbers of labeled cells and the reduced extraction of functionally active neurons are seen by 11 dpi and beyond (Fig. 6E, G, Supplementary Fig. 5). This shows RV-induced cytotoxicity in the late time window. Because our 2-photon imaging window placement is limited to hippocampal CA1 (Fig. 6), we were not able to image the activity of neurons at the distal retrogradely labeled sites. However, we examined retrogradely labeled neurons in distal sites in the perfused mouse brain at 13 days post RV infection. We were able to observe presynaptic neurons from within CA3 regions, and they appear to have healthy morphology. Overall, the in vivo RVΔG GCaMP7f-mediated calcium imaging provides a promising potential for circuit-based neural activity imaging in live animals for an applicable time window, before viral toxicity affects cell health.

### Neural circuit mapping and automated measurement of infected neurons

**Fig. 7:**
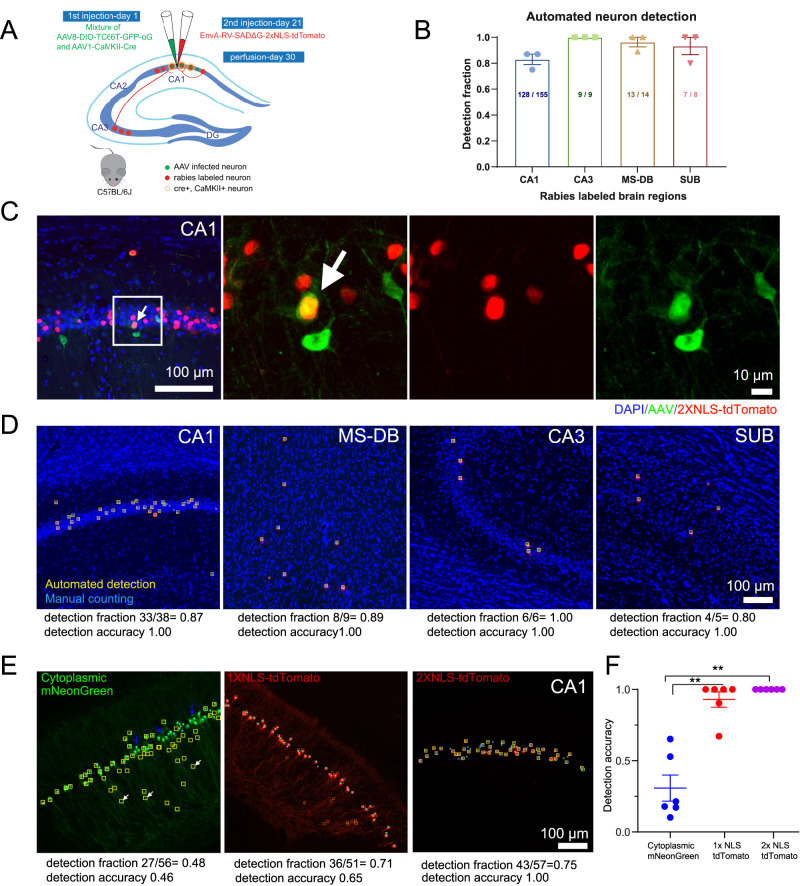
Neural circuit mapping and automated counting of recombinant rabies virus labeled neurons. **A** Schematic showing RV-2XNLS-tdTomato rabies tracing in hippocampal CA1 excitatory neurons. A mixture of AAV8-DIO-TC66T-GFP-oG and AAV1-CaMKII-Cre was injected into the hippocampal CA1 region of C57/BL6J mice, followed by rabies EnvA-SAD-ΔG-RV-2XNLS-tdTomato injection at the same site. AAV-infected neurons are shown in green, and rabies-infected neurons are shown in red. **B** Quantitative measurement of automated neuron detection fraction across multiple rabies-labeled brain regions. The data is presented as mean ± SEM, n = 3 mice. The detection fraction is defined as the proportion of neurons detected over the total number of neurons. **C** Fluorescent image of the hippocampal CA1 injection site. Neurons displaying both AAV and rabies infection are identified as starter cells. Brain slices are counterstained with DAPI, shown in blue. **D** Examples of 2XNLS-tdTomato rabies-labeled brain regions and automated detection in CA1, MS-DB, CA3, and SUB regions. Automated detection is represented by yellow square boxes, and manual detection is represented by blue asterisks. Detection fraction and detection accuracy are provided below the images. The detection accuracy is defined as the correct detection number over the total detection number. **E** Examples of automated neuron extraction using three different rabies strains: cytoplasmic mNeonGreen, 1XNLS-tdTomato, and 2XNLS-tdTomato. Detection fraction and detection accuracy are shown below the images. **F** Quantitative comparison of automated extraction in samples shown in (**E**).

To show the newly generated recombinant RVs for enhanced neural circuit mapping applications and, more broadly, for usage in animal models, we injected recombinant rabies virus expressing tdTomato coupled to nuclear localization signals for automated neuron counting (Fig. 7). The experimental design is presented in Fig. 7A. Briefly, two helper AAVs expressing: (1) TC66T (TVA variant, EnvA receptor) [37], oG (rabies virus glycoprotein), and GFP in a Cre-dependent manner and (2) Cre under the control of CamKIIa promoter; were injected in the CA1 region of the hippocampus of three C57/BL6J mice. After 21 days, EnvA-pseudotyped RVΔG 2xNLS-tdTomato was injected in the same location. Thirty days after AAV injection, mice were perfused and brains were processed for imaging. Neurons in the CA1 produce both cytoplasmic green (AAV) and nuclear red (RV) fluorescent signals (Fig. 7C). Co-infected neurons are considered starter neurons allowing for monosynaptic retrograde spreading of the rabies virus. The entire scanning of the brain allows for the detection of positive cells with red fluorescent nuclear signal in CA1, medial septum and diagonal band (MS-DB), CA3, and subiculum (SUB) (Fig. 7D). The connectivity strength and pattern revealed by the new rabies variant is consistent with our previous study [38]. Automated neuron measurement produces a high detection rate with values of 82.5%, 100%, 92.9%, and 87.5% in CA1, CA3, MS-DB, and SUB, respectively (Fig. 7B). We compared different fluorescent reporters for automated neuron detection: RVΔG mNeonGreen (cytoplasmic green signal), RVΔG 1X-NLS tdTomato (red nuclear signal with cytoplasmic leakage) and RVΔG 2XNLS-tdTomato (red nuclear signal); example detection images are presented in Fig. 7E. Data from the quantification of 6 replicates of the experiments is presented in Fig. 7F. RVΔGs with nuclear-localized reporters (RVΔG 1xNLS-tdTomato, and RVΔG 2xNLS-tdTomato) produce minimal neurite labeling, thereby facilitating the accurate identification of rabies labeled somata despite some spatial location overlap between them. Compared with cytoplasmic RVΔG mNeonGreen labeling, RVΔG 1xNLS-tdTomato and RVΔG 2xNLS-tdTomato labeling allows for improvements in software-based, automated neuron detection analysis (Fig. 7F). Under our testing conditions, quantitatively 1xNLS and 2xNLS rabies viral labeling has much higher rates of correct detection (mean proportion of correct detection: RVΔG mNeonGreen, 0.31, 1xNLS, 0.92, 2xNLS, 1.0; mNeonGreen vs. 1xNLS, *p* = 0.0022, mNeonGreen vs. 2xNLS, *p* = 0.0022, Mann Whitney tests). Altogether these data confirm the validity of our new recombinant rabies viruses for brain-wide neural circuit mapping in rodent models and support they provide useful tools for automated tracking and quantification of infected neurons in brain sections.

#### Correlative light, X-ray, and electron microscopy of RV-labeled cells

**Fig. 8:**
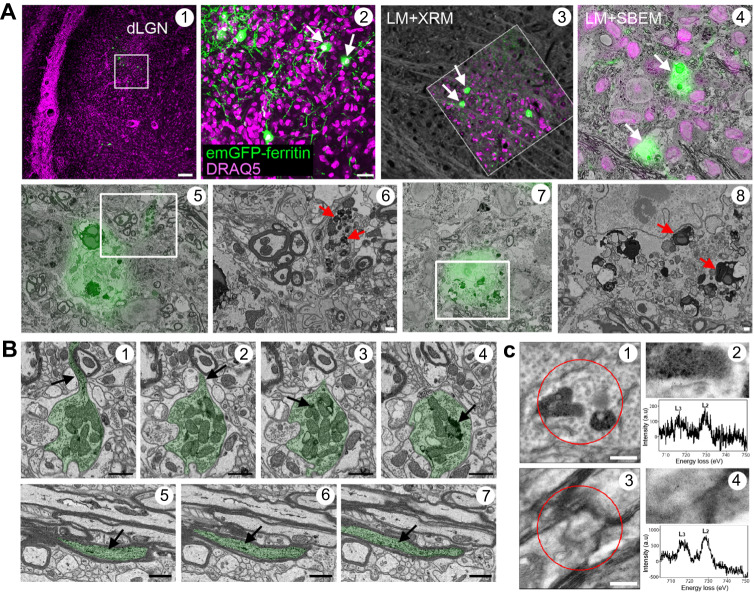
RVΔG-mediated, emGFP-ferritin cellular labeling enables multi-modal multi-scale correlative 3D light, x-ray, and electron microscopy (CLXEM). **A1**, **2** Low- and high-power confocal images showing EnvA-pseudotyped RVΔG emGFP-T2A-MLS-FTL labeled neurons and their neurites (green) and DRAQ5 labeled nuclei (magenta) in in the lateral geniculate nucleus (dLGN). DRAQ5 labeling is used to co-register confocal and XRM data. Scale bar, 50 or 10 µm. **A3** Overlay of an XRM slice on a confocal image to show locations of neurons expressing emGFP-ferritin (white arrows). **A4** An SBEM slice registered with the emGFP-ferritin confocal image plane. **A5**, **6** Overlay of emGFP-ferritin labeling in a target neuron and dendrite (box). Magnification of image (**A6**) shows ferritin particles as highly accumulated electron-dense materials inside of the dendrite (red arrows). **A7**, **8** Overlay of emGFP-ferritin labeling in another target neuron soma (box) and ferritin accumulated electron-dense materials are shown in cytoplasm (**A8**). Scale bar: 1 µm. **B** Electron microscopy (EM) examination of a different case with direct injection of un-pseudotyped RVΔG emGFP-T2A-MLS-FTL in the dLGN. **B1**–**7**. Series of electron micrographs across a labeled axon terminal (**B1**–**4**) and a labeled myelinated axon (**B5**–**7**). Ferritin particles (black arrows) are scattered in the axon (**B1**, **2** and **B5**–**7**) near a synaptic terminal, and the ferritin aggregates are pronounced near mitochondria of the presynaptic bouton (b3,4). Scar bar, 1 µm. **C** The background-subtracted electron energy loss spectrum (EELS) acquired from an RVΔG emGFP-T2A-MLS-FTL infected neurite (**C1**, **2**) and axon (**C3**, **4**). The EELS spectra (**C2**, **4**) show Fe L2,3 edge signals (edge onset occurring at 708 eV). To increase the signal-to-noise ratio, the spectrum in Fig **C1** is summed across five individual measurements.

As brain neural circuit mapping becomes more comprehensive, it is important to investigate the connection between neurons and understand neuronal biology on a cellular micro-scale [39, 40]. EM is used to study brain cells at the organelle and macromolecule scale, showing ultrastructural details of the specific biology of neurons. Recombinant RVs expressing ferritin light chain were generated as reporters for electron microscopy and a correlated light, X-ray, and electron microscopy workflow (CLXEM) [41] was applied to evaluate the utility of these RV reporters for ultrastructural mapping of specific neuronal populations (Supplementary Movie 4), using the labeled V1-projecting dLGN cells as the test case. Since EM image quality of samples prepared for correlated fluorescence microscopy and EM can be compromised by weaker chemical fixatives, we added a small amount of glutaraldehyde (0.1%) to the sample preparation to better preserve ultrastructure that also allows for parallel high-quality fluorescent light microscopy (LM) mapping by confocal microscopy in the same sample. For precise image alignment across scales, we added fiducial reference labels to cell nuclei, which can be seen across all three imaging modalities [41]. Fiducial reference labeling is accomplished by staining nuclei with the DNA intercalating dye DRAQ5 [42], thus providing distributed and easily detected landmarks by labeling all cell nuclei throughout the entire 100 µm thick brain slice. RV infected and ferritin-expressing neurons and fiducial DRAQ5 labeling were first confirmed by confocal fluorescence microscopy (Fig. 8A, 1–2). To improve the alignment accuracy across imaging fields of view that include the RV-ferritin infected target cells, we carried out microCT -X-ray microscopy imaging (XRM) (Supplementary Movie 4). The XRM imaging of tissue and cell structures within the dLGN allows visualization of fine structures as small as nucleoli and correlate DRAQ5 labeling fluorescence with the sample after osmification and epoxy embedding. The two RV-ferritin infected neurons (arrows, Fig. 8A3) are targeted for higher resolution 3D EM analysis. After identification of regions of interest and imaging by serial block-face scanning EM (SBEM), the registration of confocal data with the final SBEM volume was performed to align RV-ferritin infected target cells within the final SBEM volume (Fig. 8A4). The somata and neurites of RV-ferritin infected neurons show electron-dense (putative ferritin) accumulations (Fig. 8A, 5–8). Ferritin particle accumulations can also be seen in axon terminals (Fig. 8B, 1–4) and a myelinated axon (Fig. 8B, 5–7) labeled by RVΔG emGFP-T2A-MLS-FTL. Note that ferritin particles (arrows) are scattered within the axon (Fig. 8B, 1–2 and 5–7) proximal to a synaptic terminal and are at higher density near mitochondria of presynaptic boutons in this image. In each case, electron-dense ferritin particles are easily distinguishable, and axons with ferritin labels are readily identified as distinct from non-infected axons (Fig. 8B, 5–7).

The putative ferritin particle densities seen in SBEM images may have resulted from the potassium ferrocyanide of reduced osmium tetroxide, which is typically required for the standard high-contrast *en bloc* staining of SBEM sample preparation. The potassium ferrocyanide contains iron, which may confound iron signals from the ferritin particles. Thus, we tested for the presence of iron to confirm the element source of these visualized particulate densities with RV infected brain slices post-stained with only 2% osmium tetroxide. Iron containing ferritin particles in the RVΔG emGFP-T2A-MLS-FTL infected area of dLGN was confirmed using electron energy loss spectroscopy (EELS) [43] (Fig. 8C, 1, 2). The background-subtracted EELS acquired in RV-ferritin infected neurite (Fig. 8C, 1, 2) and axon (Fig. 8C, 3, 4) and the EELS spectrum (Fig. 8C, 2,4) reveal the characteristic peaks of iron (Fe) L2,3 edge signals (edge onset occurring at 708 eV). The EELS spectra were measured from the region outlined by the red circle (Fig. 8C, 1,3) with a CCD detector set to an exposure time of 15 s. These results confirm that the electron density seen in these images is from iron and not from aggregates of other heavy metal atoms.

## Discussion

Earlier generations of recombinant rabies viruses have been useful for neural circuit analysis across a variety of species. Here, we generated 20 new recombinant RV reporters that substantially expand RV’s capabilities. The new variants include expression of improved fluorescent proteins (e.g., tdTomato, mNeonGreen and “fluorescent timer”) and functional reporters (e.g., nanoluciferase and ferritin). The rabies viral expression of reporters can be targeted to specific sub-cellular locations and organelles for broad utility. We showcase this new set of rabies viral tools that expand our circuit mapping repertoire to detailed 3D LM and X-ray microscopy and finally to ultrastructural EM analyses. Notably, the new RV variants work across multiple scales and different imaging modalities and expand neuroscience applications to those not possible with earlier generation reagents. Luciferase is useful for functional neuroscience studies, particularly those investigating light sensitive tissues [21, 22].

Our new fluorescent RVΔG reporters, including variants that target fluorescent markers to specific neuronal subcellular locations, show intense neuronal labeling in vivo in diverse brain regions including somatosensory and visual cortex, hippocampal formation, visual thalamus and the retina (Fig S4). These new variants significantly enhance rabies-virus-mediated cell labeling and circuit mapping approaches. RVΔG-1xNLS-tdTomato and RVΔG-2xNLS-tdTomato can be used to facilitate automated quantification of rabies labeled cells. RVΔG ChR2-EYFP-Kv is designed to target expression of ChR2-EYFP to the neuronal soma and proximal dendrites. This improved targeting can achieve cellular resolution circuit mapping with focused excitation of soma-targeted ChR2 [33]. RVΔG-PSD95-mNeonGreen and RVΔG SNPN-tdTomato are designed to highlight labeling of postsynaptic dendritic spines, and presynaptic axon boutons, respectively. Since the RVΔG reporter construct can be combined with cytoplasmic or other specific reporters using an autocatalytic protein cleavage sequence or using a second ORF in the genome of RV [8, 44], we can multiplex different combinations to further expand our RVΔG reporter repertoire, such as RVΔG-mNeonGreen-T2A-2xMito-dTomato. Care must be taken for multiplex strategies to use labels with good spectral separation.

Because RVΔG viruses can replicate their genomes, infected neurons express large quantities of fluorescent proteins. This gives our new RVΔG variants unique advantages of fast and strong labeling. In contrast to slow and weak AAV-mediated expression in neuronal cultures (~1 week), our new RVΔG variants intensely label SCG cell bodies, axons or mitochondria that are sufficiently bright to image at 24 h post infection. We tested new RVΔG variants in cultured SCG neurons and verified the utility of RVΔG Slow-FT. This type of “timer” reporter can be used to follow the cellular spatial migration of specific proteins. A chimeric fusion between timer proteins and a protein of interest would appear blue early after infection, then would turn red over time. This allows tracking of the spatial distribution and temporal expression of specific proteins can be determined by using imaging analyses. Using the same model, RVΔG PSD95-mNeonGreen produces bright labeling to show detailed somatic and dendritic structures. Moreover, RVΔG 2XMito-dTomato is very useful for labeling neuronal mitochondria by visualizing their morphologies and motility. One striking advantage of RVΔG 2XMito-dTomato over commonly used mitochondria-selective chemical dyes is the absence of high background noise. We foresee that these new RVΔG variants will have broad applications in neuronal culture experiments.

The proof-of-concept applications of these tools we documented show the potential of their discovery power. We documented detailed microstructural changes of rabies-labeled neurons in aging and Alzheimer’s disease mouse models, including dendritic spine loss and reduced mitochondrial size in labeled cortical and hippocampal excitatory neurons with RVΔG-PSD95-mNeonGreen and RVΔG 2XMito-dTomato.

Since RVΔGs are retrograde viral tracers, they allow neurons to be targeted on the basis of their connectivity. They can also be targeted to specific cell types through EnvA-pseudotyping and expression of the EnvA receptor, TVA, in defined cell types. We leveraged these properties to demonstrate the utility of our new RV vectors for neural circuit mapping via expression of ferritin and EGFP/emGFP. For proof of principle, we showed that our rabies viral vectors that encode GFP and ferritin enable multi-scale circuit analysis by electron microscopy (EM) and optical imaging. With further improvements in sample preservation and preparation, we foresee that high-resolution mesoscale circuit mapping and detailed ultrastructural analysis of a sub-volume by EM in the same experimental animals will be possible. Similar results were published using vesicular stomatitis virus (VSV), another member of the rhabdovirus family [45].

RVΔG is an excellent tool for in vivo neural circuit mapping. Its biology and structure allow us to pseudotype the virus with the glycoprotein from a different virus, such as EnvA- pseudotyped RVΔG. In conjunction with helper AAVs expressing TVA, the receptor associated with EnvA to allow for viral entry, EnvA-pseudotyped RVΔG enables monosynaptic input tracing from specific neuron types in a defined time window. Other viral tools for neural circuit mapping have not yet achieved the same level of tropism specificity. RVΔG induces significant cytotoxicity in the mammalian brain within 2 weeks post-infection [4]. Thus an important goal is to further reduce viral toxicity through ongoing and future work. Low-toxicity rabies viral vectors are critically required for longitudinal imaging to monitor normal circuit development and disease progression, as well as for delivering functional payloads in targeted neural circuit elements in the brain. Some investigators have attempted to attenuate this toxicity by modifying and/or deleting viral genes expressed by these rabies virus vectors (e.g., [6, 7]). Ideally data collection with further improved RV tools will last more than several weeks or months for the collection of high-quality, longitudinal data.

Currently, community access to resources of new genetically-modified neurotropic viruses is limited. Our new RV resources (all versions of un-pseudotyped and EnvA-pseudotyped RVΔGs) will be available to the neuroscience community through our established service platform at the UC Irvine Center for Neural Circuit Mapping (https://cncm.som.uci.edu/). Our distribution platform includes a mechanism for user feedback and user suggestions for further technology improvements and resource generation. We anticipate that our work will expand the availability of authenticated new viral-genetic tools for mapping anatomical and functional connectomes of defined neuronal types in the brain of multiple species to the neuroscience community.

## Materials and Methods

### Animals

All experiments were conducted according to the National Institutes of Health guidelines for animal care and use and were approved by the Institutional Animal Care and Use Committee (IACUC), protocol number AUP-22-163, and the Institutional Biosafety Committee (IBC), protocol number BUA-R100, of the University of California, Irvine (UCI). All mice used for rabies in vivo testing have the same C57BL/6J background and were at least 8 weeks of age. Both male and female mice were used for the experiments. All strains used in this study were originally purchased from the Jackson Laboratory and breeding is maintained in Dr. Xiangmin Xu’s laboratory vivarium at UCI. Strains used in this study include: C57BL/6 J (JAX stock#: 000664) and 5xFAD (MMRRC Strain #034848-JAX) (MODEL-AD at UCI), and B6 PV-Cre (JAX stock#: 017320).

### Cloning and generation of recombinant rabies viruses from cDNA

The template for the initial cloning was pSADdeltaG-F3, a gift from Edward Callaway (Addgene plasmid # 32634) [2]. The plasmid was digested with restriction endonuclease NheI, and inserts were cloned following ligation using T4 DNA ligase (New England Biolabs). Fluorescent reporters were amplified from the plasmids listed in Table S1. Inserts were amplified by PCR to add restriction sites to their termini and subcloned under a CMV promoter in a vector harboring the necessary added sequences (localization signals). Subclones were transfected into HeLa cells to validate the correct fluorescence and localization of the fluorescent proteins.

B7GG cells were plated in a 6-well plate. The following day they were transfected with 3 µg of plasmid harboring the RV genome, 1.5 µg of pcDNA-SADB19N, 750 ng of pcDNA-SADB19L, 750 ng of pcDNA-SADB19P and 500 ng of pcDNA-SADB19G using Lipofectamine 2000 (ThermoFisher, 11668019). These plasmids were a gift from Edward Callaway (Addgene plasmid # 32630, 32631, 32632, 32633) [14]. After reaching confluency, cells were passaged and growth was carried out until >70% of the cells were infected. Virus supernatant was collected and filtered consistently every 2–3 days through a 50 ml 0.45um vacuum filter (SE1M003M00, MilliporeSigma™). The filtered virus supernatant was stored at −80 °C until the amplification step described later.

### In vitro characterization of recombinant rabies viruses

Initial fluorescence and localization controls were performed in HeLa cells. These cells were grown in Dulbecco’s Modified Eagle Medium (DMEM; Gibco, 12800017) supplemented with 1X Antibiotic Antimycotic solution (Omega Scientific, AA-40) and 10% NCS (Omega Scientific, NC-04). RVs were grown using B7GG cells [2] in DMEM (Gibco, 12800017) supplemented with 1X Antibiotic Antimycotic solution (Omega Scientific, AA-40) and 2% FBS (Omega Scientific, FB-12). Infected cells were grown until confluency and passage every 2 days. For imaging in vitro samples, cells were plated on glass coverslips and grown as previously described. Cells were fixed for 20 min in PBS + 3.7% formaldehyde, washed three times in PBS for 5 min, permeabilized 10 min in PBS + 0.2% Triton X-100, washed three times in PBS for 5 min, nuclei were stained with DAPI (4 µg/ml) for 2 min, and washed once with PBS before being mounted. Immunofluorescent staining was performed using antibodies against nanoluciferase (R & D systems; 965853) or Ferritin (ab69090; Abcam). Luminescence assays were performed using Nano-Glo Luciferase Assay (N1110; Promega) according to the manufacturer’s instructions.

Superior cervical ganglia (SCG) were isolated from embryonic day-16–17 Sprague-Dawley rat embryos (Charles River) and neurons were cultured in tri-chambers as described previously [46]. Briefly, the SCG were incubated in 250 µg/ml of trypsin (Worthington Biochemicals) for 15 min. followed by 2x washing in HBSS buffer. Prior to plating, the ganglia were triturated in complete neuronal media using a fire-polished Pasteur pipette and then 2/3 of a ganglion was plated in the soma (S)- or compartments of the Teflon ring. The Teflon ring was placed within either a 35-mm plastic tissue culture dish or optical plastic dish (Ibidi) coated with 500 µg/ml of poly-DL-ornithine (Sigma-Aldrich) diluted in borate buffer and 10 µg/ml of natural mouse laminin (Invitrogen). Neuronal culture medium consists of neurobasal medium (Gibco) supplemented with 100 ng/ml nerve growth factor 2.5S (Invitrogen), 2% B27 (Gibco), 2 mM glutamine (Invitrogen), penicillin, and streptomycin. Two days after plating, neuronal cultures were incubated with 1 mM of the antimitotic drug cytosine-D-arabinofuranoside (AraC; Sigma-Aldrich) for 2 days to kill dividing non-neuronal cell types. Neurons were cultured for 14–21 days prior to experiments. RV inoculum (10^4^–10^5^ pfu) was added in the S-compartments. Depending on the assay, infected axons or soma were imaged either at indicated hours post infection (hpi). Live-cell imaging was performed on a DMi8 inverted epifluorescence microscope (Leica) equipped with a DFC9000GT camera (Leica). Neuron cultures were kept in a humidified stage-top incubator (Tokai Hit) at 37 °C with 5% CO_2_ during imaging. The movies and images were prepared using Leica imaging software and Adobe Photoshop.

Sytox Green Nucleic Acid Stain (Invitrogen S7020) was diluted 1:100 in DMEM, and 3 ul was added into the S compartments (final concentration is 500 nM). After 30 min incubation, media was replaced and S-compartments were imaged in phase, green and red channels. The ratio of green and red double positive neurons (dead RV infected) to red only neurons (RV infected) was calculated to determine the neurotoxicity.

### Amplification, pseudotyping, and concentration of viral stocks

The protocols for rabies recovery, expansion, concentration, and titer have been described previously [14, 47]. Virus supernatant collected from the transfection assay was used for the expansion of viral stocks. Recombinant RV was grown using B7GG cells in DMEM supplemented with 2% fetal bovine serum (FBS) (SH30070.03, Cytiva) in 37 °C, >80% humidity, 5% CO_2_. B7GG cells were grown to 50% confluency in ten tissue culture dishes (353025, Falcon™) and infected with virus supernatant. Three days post-infection, the cells were checked for fluorescence and supernatant was collected and filtered through a 0.45 μm sterile vacuum filter unit (S2HVU02RE, MilliporeSigma). The supernatant was collected every 2–3 days for up to four collections depending on cell health. The filtered supernatant was stored at −80 °C until pseudotyping and ultracentrifugation steps. Only supernatant collected from plates with >80% infection was used for ultracentrifugation to maximize the titer of the final concentrated stock for in vivo tests.

Recombinant RV was grown in BHK-EnvA cells (a gift from Edward Callaway) to produce rabies virions with an EnvA coat protein. BHK-EnvA cells were grown in DMEM supplemented with 2% FBS in 37 °C, >80% humidity, 5% CO_2_. BHK-EnvA cells were grown to 50% confluency on 10 tissue culture dishes with grids (353025, Falcon™) and infected with virus supernatant. The cells were rinsed three times with 1x Dulbecco’s phosphate-buffered saline (DPBS) (14190136, Gibco™) and trypsinized with pre-warmed 0.25% trypsin-EDTA (25200056, Gibco™) to remove any unpseudotyped virus 1 day post-infection. Three days post-infection, the cells were checked for fluorescence and virus supernatant was collected and filtered as stated previously in the amplification step. Only supernatant collected from plates with >60% infection was used for ultracentrifugation to ensure a high titer of the final concentrated stock for in vivo tests.

Both unpseudotyped and pseudotyped rabies stocks followed the same procedure for ultracentrifugation (Optima L-100XP, Beckman Coulter). Around 225 mL of frozen virus supernatant was thawed in a 35-37 °C water bath and centrifuged using SW32Ti rotor (Beckman Coulter) for 2 h at 20,000 RPM (70,000 *g*) in 4 °C. This step was repeated twice to concentrate a total volume of 450 mL. The supernatant was discarded, and each pellet was resuspended using cold 1x Hanks’ Balanced Salt Solution (HBSS) (14175095, Gibco™). The viral suspensions were combined and overlaid on top of 2.5 mL of 20% sucrose/1x HBSS before ultra centrifugation using the SW55Ti for 2 h at 21,000 RPM (50,000 *g*) in 4 °C. The final pellet was resuspended in 100 μL of cold HBSS, and the concentrated viral stock was stored in the −80 °C prior to viral titer determination and in vivo testing.

HEK293T-TVA800 cells (a gift from Edward Callaway) were used to titer both unpseudotyped and EnvA-pseudotyped rabies. Titration were performed using two different protocols: Using Chamber slides: A 8-well chamber slide (354118, Falcon™) is seeded at a density of 1.5 × 10^4^ cells per well, and a ten-fold serial dilution is prepared from 10^3^ to 10^9^. Each well is infected with a dilution. Three days post-infection, the chamber slides are scanned using an inverted microscope. The images are stitched and manually counted to determine the number of infected cells. Generally, wells containing 10–150 fluorescent cells are counted. Titer calculations are performed following the existing protocol described in [14]. Viral titers for unpseudotyped rabies viruses were consistently between 10^9^ and 10^10^ infectious units (IU)/mL, while the viral titer for EnvA-pseudotyped rabies viruses were consistently one order lower, between 10^8^ and 10^9^ IU/mL. Using FACS: A 12-well plate (3513, Corning™) is seeded at a density of 1.5 × 10^5^ cells per well. A serial dilution was prepared, and the cells were infected with applied virus volumes of 2.5 μL, 0.25 μL, 0.025 μL, 0.0025 μL, 0.00025 μL (Log 0, 1, 2, 3, 4 respectively). Three days post-infection, the cells were dissociated using pre-warmed 0.25% trypsin-EDTA (25200056, Gibco™) and fixed with cold 2% paraformaldehyde (PFA). The cells were filtered using a 5 mL round-bottom polystyrene test tube with cell strainer snap cap (352235 Falcon™). A flow cytometer (MA900, SONY) was used to analyze the percentage of infected cells for each log dilution. Generally, only logs 1, 2, and 3 are used for the calculation. Titer calculations were performed following the existing protocol described in Wickersham et al. [47]. Viral titers for unpseudotyped rabies viruses were consistently between 10^9^ and 10^10^ infectious units (IU)/mL, while the viral titer for EnvA-pseudotyped rabies viruses were consistently 1 log lower, between 10^8^ and 10^9^ IU/mL.

### In vivo mouse viral injections

The general viral injection procedure has been previously described [10, 48]. Both AAV and rabies virus were injected following the same procedure. The mice were anesthetized and a digital stereotaxic instrument guided by a digital atlas (Angle Two™ Stereotaxic for Mouse) was used to target the following coordinates: S1BF (primary somatosensory cortex, barrel field): anteroposterior (AP) = −0.70 mm, lateromedial (ML) = ± 2.85 mm, and dorsoventral (DV) = −1.85 mm; dSUB (dorsal subiculum): AP = −3.40 mm, ML = ± 1.96 mm, and DV = −1.67 mm; dCA1 (dorsal hippocampal CA1): AP = −1.94 mm, ML = ± 1.40 mm; and DV = −1.35 mm; DLGN (dorsal lateral geniculate nucleus): AP = −2.30 mm, ML = ± 2.14 mm, and DV = −2.75 mm; V1B (primary visual cortex, binocular area): AP = −3.40 mm, ML = ± 2.70 mm, and DV = −1.40 mm; V1M (primary visual cortex, monocular area): AP = −3.40 mm, ML = ± 1.96 mm, and DV = −1.00 mm. Virus was loaded into a pulled glass capillary (#BF100-50-10, Sutter Instrument) (tip inner diameter of 20–30 μm), and virus was injected using a pressure-controlled microinjection dispense system (picospritzer III, Parker Hannafin) at 20–30 nL/min with 10 ms pulse duration. A total volume of 200–400 nL was injected into each targeted region depending on the size of the targeted area.

Unpseudotyped SAD-B19-RVΔG recombinant viruses were directly injected into C57BL/6 J background mice without the use of helper viruses, whereas a combination of AAVs expressing Cre, TVA, and G were used to transcomplement EnvA-pseudotyped SAD-B19-RVΔG to identify monosynaptic inputs. For the EM experiments, AAV2-retro-syn-Cre (1.57E + 13) was injected into V1B while AAV8-DIO-TC66T-2A-mCherry-2A-oG (2.62E + 13) was injected into DLGN and incubated for 21 days followed by the injection of EnvA-SAD-B19-RVΔG-emGFP-T2A-MLS-FTL into the DLGN. The mice were perfused after an additional 9 days.

### Histology and immunochemical staining of mouse brain tissue

Mice were transcardially perfused with 1x phosphate buffered saline (PBS) followed by 4% paraformaldehyde (PFA), and their brains were dissected and post-fixed in 4% PFA for 24 h. Samples were transferred to 30% sucrose/1x PBS. The brain tissue was frozen with dry ice and sectioned coronally into 30-μm-thick slices using a sliding microtome (SM2010R, Leica). Select brain sections expressing FTL were stained with Anti-Ferritin Light Chain antibody (ab69090, ABCAM, 1:500 dilution) followed by Cy™5 AffiniPure Donkey Anti-Rabbit IgG (H + L) (Code: 711-175-152, Jackson ImmunoResearch, 1:200 dilution). Brain sections expressing PSD95 were stained with PSD95 Rabbit mAb (A0131, ABcolonal, 1:500 dilution) followed by Cy™5 AffiniPure Donkey Anti-Rabbit IgG (H + L) (Code: 711-175-152, Jackson ImmunoResearch, 1:200 dilution). All sections except the ones stained with AmyloGlo were counterstained with 10 μM 4,6-diamidino-2-phenylindole (DAPI) and mounted for imaging. Amylo-Glo RTD amyloid Plaque Stain Reagent (Catalog: TR-300-AG, Biosensis) was used following the manufacturer’s protocol to visualize amyloid beta plaques, which are characteristic in the 5xFAD Alzheimer’s disease model mice. Sections stained with AmyloGlo were counterstained with 3 μM DRAQ7™ (Catalog: 7406, Cell Signaling Technology) and mounted for imaging. Fluoromount-G (Catalog: 0100-01, SouthernBiotech) was the mounting media used.

### Imaging and analysis

Sections with sparse neuronal labeling were imaged using a confocal microscope (FV3000, Olympus) under 20x dry and 60x oil objective lens to characterize viral labeling in neurons. Tile scans were stitched and *z-*maximum projection (2D) images were generated using the Olympus confocal software. All files were exported as TIFF format. Brain sections expressing SAD-ΔG-1xNLS-tdtomato and SAD-ΔG-2xNLS were imaged using an automated slide scanning system (VS120, Olympus) under a 10x objective and were stitched and converted to TIFF format for automated counting analysis described later.

For mitochondria image processing and analysis, cell fluorescence intensity measurements were performed by using the Fiji-ImageJ software analysis tools. 2D images were preprocessed using the following commands: (1) “subtract background” to remove background noise; (2) “adjust threshold”; (3) “make into binary image”; and (4) perform analysis of mitochondria morphology. The binary images were used as the input for the “analyze particles” command, measuring for “area” and “perimeter”. The area (μm^2^) was used for data reporting and statistical analysis. Particles less than 0.1 μm^2^ or larger than 2.4 μm^2^ were excluded for statistical analysis.

### Neural circuit mapping and automated counting of infected neurons

To map the neural circuit in mouse hippocampal CA1, we used the following AAVs: AAV8-DIO-TC66T-2A-GFP-2A-oG (Salk Institute, CA, US, 5.06×10^13^ GC/ml) and pENN.AAV.CaMKII 0.4.Cre.SV40 (Addgene viral prep #105558-AAV1, 5.3 × 10^13^ GC/ml). pENN.AAV.CaMKII 0.4.Cre.SV40 was a gift from Dr. James M. Wilson. The AAV8-DIO-TC66T-2A-GFP-2A-oG was 1:2 diluted with phosphate-buffered saline (PBS). The pENN.AAV.CaMKII 0.4.Cre.SV40 was 1:4 diluted with PBS. These two diluted helper AAVs were then 1:1 mixed. Finally, 0.1 μl of the diluted AAV mixture was injected into the CA1 target region on day 1. After 21 days, the mice were injected with the rabies virus EnvA-SADΔG-RV-2xNLS-tdTomato (4.1 × 10^7^ IU/ml, 0.4 μl) at the same injection site using pressure injection. The viruses were injected into the pyramidal layer of dorsal hippocampal CA1 using the following coordinates: anteroposterior (AP) −1.94 mm, mediolateral (ML) −1.40 mm, dorsoventral (DV) −1.35 mm, all values given relative to bregma.

To perform objective evaluation of the labeling quality, we built a custom-made multi-layer convolutional neural network (CNN) to perform automatic neuron detection with the captured microscopy images. We manually pick up 1512 neuron patches and 2348 background patches from 1xNLS red channel, 2xNLS red channel, and the reference green channel of 1xNLS data. Each extracted neuron or background patch has 56*56 pixel in size, and only contains the color channel in which the neurons are labeled. Z-score operation is taken to each patch for intensity normalization purpose, and pixels with negative intensity clipped to 0. In order to fit the network input requirement, the patches are converted to 3-color channels images in which the red channel contains the original patch.

The custom-made multi-layer CNN is built with the Deep Learning toolbox of MATLAB 2020b and contains 12 layers. The first layer is the input layer. Following layers contain 2 convolution layers with 14*14 and 7*7 weights, respectively. Each convolution layer accompanies by a batch normalization layer, a max pooling layer, and a ReLU activation layer. The last three layers contain a fully connected layer that generates two outputs corresponding to the neuron and background class, a SoftMax layer and a classification layer. Detailed explanation of the layers’ structure and features can be found in their corresponding studies [49–52]. During training, 900 neuron patches and 900 background patches are randomly selected as training data set, while the remaining data are served as validation data set. Ten training epochs are used and learning rate is set as 0.001. Training ends with validation accuracy at 99.53%. During actual neuron detection, each pixel in the microscopy image (except the 1–28 pixel close to each edge) will be used to construct a 56*56-pixel patch, with the pixel located at center, and have its intensity z-scored. The CNN will provide 0 if the patch is classified as background, and provide 1 if the patch is classified as neuron. In this way the image will be converted to a binary mask in which intra-neuron pixels are distinguished from background. Individual neurons will be isolated from the binary mask.

### In vivo live imaging of neurons activity

To validate RV-GCaMP7f infected cells’ response to intracellular calcium level increase, cultured SCG cells were infected with the unpseudotyped SAD-B19-ΔG-RV-GCaMP7f virus. Two days post-infection, the SCG cells were imaged before and post 50 mM KCl treatment with a live imaging fluorescent microscope (Leica DMi8).

To further test in vivo monitoring of neuronal activities in awake animals, excitatory CA1 neurons in C57 mice were labeled with EnvA-pseudotyped RVΔG GCaMP7f. The mouse was injected with 0.1 μl of helper AAV mixture. The AAV mixture contained the 1: 1 mixture of AAV8-DIO-TC66T-2A-mCherry-2A-oG (titer: 3.42 × 10^13^ GC/ml) and AAV1-Camk-Cre (titer: 1.9 × 10^12^ GC/ml). 21 days after the AAV injection, 0.2 μl of EnvA-SAD-B19-ΔG-RV-GCaMP7f (titer: 1.2 × 10^9^ IU/ml) was injected at hippocampal CA1. For both AAV and RV injection, the following coordinate: AP -2.06 mm, ML 1.65 mm, DV −1.47 mm was used. The mouse was then implanted with a cylindrical cannula on top of hippocampal CA1 following a published protocol [53] on the same day immediately after the RV injection. Briefly, the cortical tissue and a thin layer of corpus callosum above mouse CA1 were carefully removed, and a cannula sealed with a glass coverslip at the bottom was implanted on top of CA1. A head bar was further glued on the skull to allow for fixation on a two-photon microscope (Neurolabware, CA, US). Mouse received intramuscular dexamethasone injections before the surgery and subcutaneous carprofen daily for 3 days post-surgery. Around 8 days post-infection and surgery, the mouse was fully recovered and CA1 neurons were imaged when the mouse was awake. The two-photon imaging data was recorded and further analyzed using CNMFE-based custom-written MATLAB pipelines [54].

### Correlated light and electron microscopy

For 3D correlative light, X-ray and electron microscopy in emGFP expressed mice, mice were anesthetized with an intraperitoneal injection of ketamine/xylazine and transcardially perfused with a brief flush of Ringer’s solution containing heparin and xylocaine, followed by approximately 50 mL of 0.1% glutaraldehyde/4% paraformaldehyde in 0.15 M sodium cacodylate buffer containing 2 mM CaCl_2_ (CB, pH 7.4). The brain was post-fixed overnight on ice in the same fixative and 100 µm thick coronal sections. RV infected brain slices were collected and incubated in DRAQ5 (1:1000, Cell Signaling Technology) on ice for an hour. Confocal images of emGFP and DRAQ5 signals were collected on a confocal microscopy (Olympus FluoView1000; Olympus, Tokyo, Japan) with a 10X, 20X, and 60X oil-immersion objective lens using 488 nm and 633 nm excitation.

Confocal imaged brain slices were prepared for MicroCT and SBEM as previously described [41]. Briefly, immediately after confocal imaging, brain slices were fixed in 2.5% glutaraldehyde in 0.15 M cacodylate buffer (CB, pH 7.4) at 4°C an hour. After removing the fixative, brain slices were washed with 0.15 M CB and then placed into 2% OsO4/1.5% potassium ferrocyanide in 0.15 M CB containing 2 mM CaCl_2_ for 1 h at room temperature (RT). After thorough washing in double distilled water (ddH_2_O), slices were placed into 0.05% thiocarbohydrazide for 30 min. Brain slices were again washed and then stained with 2% aqueous OsO4 for 30 min. Brain slices were washed and then placed into 2% aqueous uranyl acetate overnight at 4°C. Brain slices were washed with ddH2O at RT and then stained with 0.05% en bloc lead aspartate for 30 min at 60°C. Brain slices were washed with ddH_2_O and then dehydrated on ice in 50%, 70%, 90%, 100%, 100% ethanol solutions for 10 min at each step. Brain slices were then washed twice with dry acetone and then placed into 50:50 Durcupan ACM:acetone overnight. Brain slices were transferred to 100% Durcupan resin overnight. Brain slices were then flat embedded between glass slides coated with mold-release compound and left in an oven at 60°C for 72 h.

The MicroCT tilt series were collected using a Zeiss Xradia 510 Versa (Zeiss X-Ray Microscopy) operated at 50 kV (80 µA current) with a x20 magnification and 0.5331 µm pixel size. MicroCT volumes were generated from a tilt series of 3201 projections using XMReconstructor (Xradia). SBEMs were accomplished using Merlin or Gemini SEM (Zeiss, Oberkochen, Germany) equipped with a Gatan 3 View system and a focal nitrogen gas injection setup. This system allowed the application of nitrogen gas precisely over the block‐face of ROI during imaging with high vacuum to maximize the SEM image resolution. Images were acquired in 2.5 kV accelerating voltage and 1 µs dwell time; 4 nm XY pixels, 50 nm Z steps; raster size was 17 k × 17 k, and Z dimension was 861 images. Volumes were collected using 40% nitrogen gas injection to samples under high vacuum. Once volumes were collected, the histograms for the slices throughout the volume stack were normalized to correct for drift in image intensity during acquisition. Digital micrograph files (.dm4) were normalized and then converted to MRC format. The stacks were converted to eight bit and volumes were manually traced for reconstruction using IMOD.

We used a custom software to align and merge the SBEM data with its corresponding 3D fluorescence counterpart into one single composite volume. It enabled us to easily track and refine landmark features shared between the two modalities while their correspondence was being progressively established.

RV infected brain slices were post-stained with only 2% OsO4 to add electron density to the ferritin precipitates for the ferritin validation. After dehydration and embedding in the Durcupan resin block, 100 nm thick sections were prepared with a Leica Ultracut UCT ultramicrotome and Diatome Ultra 45° 4 mm wet diamond knife. Sections were picked up with 100 mesh gilder copper grids (G50, Ted Pella, Inc).

Sections were loaded into a JEOL JEM 3100EF equipped with an in-column Omega Filter and operating at 200 kV. Sections were pre-irradiated at a low magnification of 100x for about 30 min to stabilize the sample and minimize contamination. All zero-loss images were acquired with a hardware bin of 4 × 4 pixels using a Ultrascan 4000 CCD detector from Gatan (Pleasanton, CA, USA). Electron energy loss spectrum (EELS) was used for the detection of iron showing the presence of Fe L2,3 edge at 708 eV and 721 eV.

## Supplementary information

Supplementary data

Supplementary movie 1

Supplementary movie 2

Supplementary movie 3

Supplementary movie 4

## Data Availability

All data generated or analyzed during this study are included in this published article (and its Supplementary Data files).
