# Supplementary material for: New rabies viral resources for multi-scale neural circuit mapping
Source: Mol Psychiatry. 2024 Feb 14;29(7):1951–67. doi: 10.1038/s41380-024-02451-6 (PMC11322437; doi:10.1038/s41380-024-02451-6)
Supplement: Supplementary file 1 — Supplementary data [file 41380_2024_2451_MOESM1_ESM.docx]

**Table S1.** List of template plasmid providing reporter genes for recombinant RV cloning


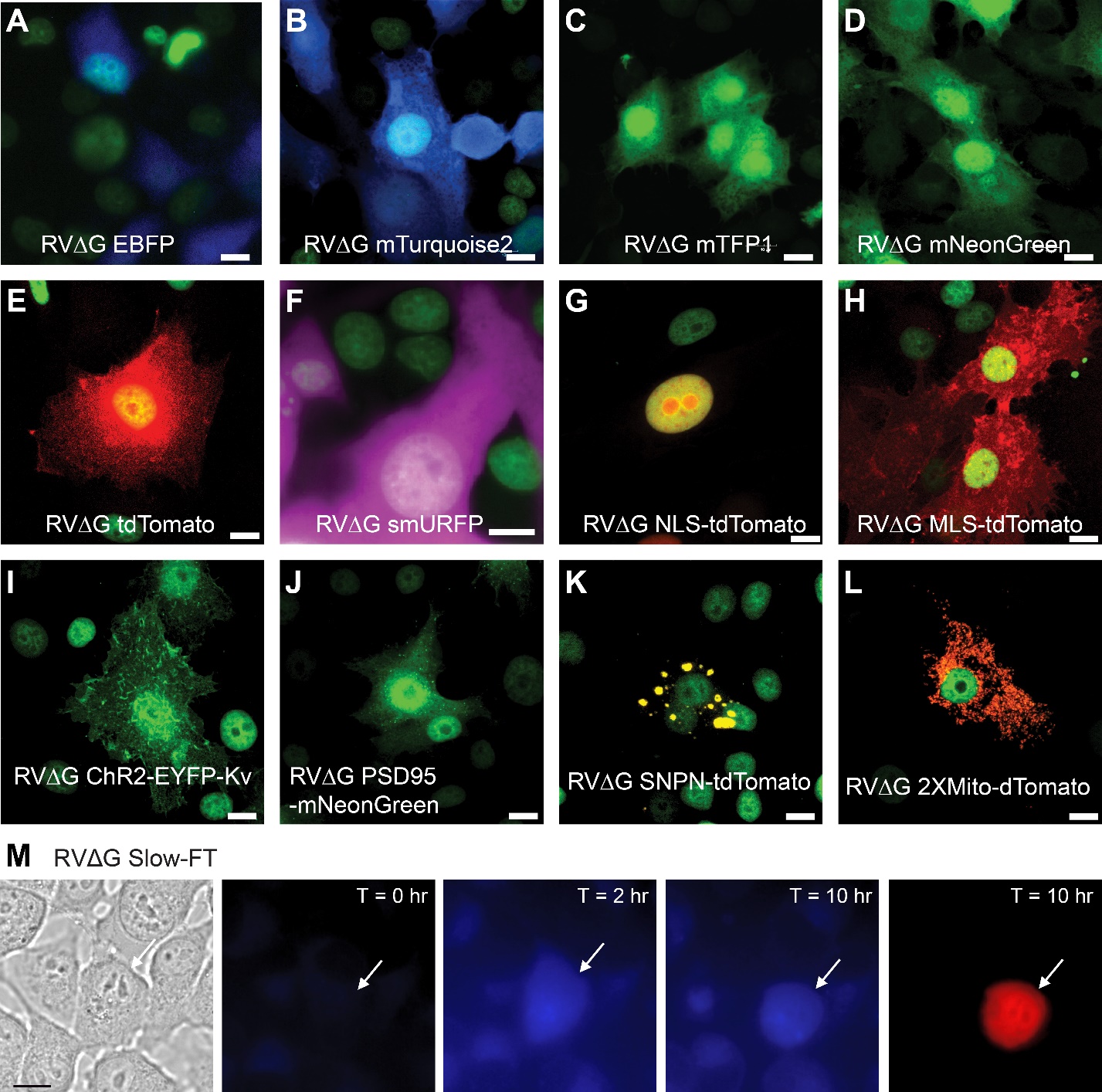


**Supplementary Figure 1: Imaging of fluorescent reporter expression by recombinant rabies viruses.**

**A to L.** Imaging of recombinant rabies viruses harboring fluorescent reporters localizing to specific cellular compartments. All B7GG cells shown express nuclear localized EGFP. Viruses were grown in B7GG cells until cells reached optimal confluency, cells were then fixed in 3.7% formaldehyde and mounted on slides. **A.** Cytoplasm targeted EBFP. **B.** Cytoplasm targeted mTurquoise2. **C.** Cytoplasm targeted mTFP1. **D.** Cytoplasm targeted mNeonGreen. **E.** Cytoplasm targeted tdTomato. **F.** Cytoplasm targeted smURFP. **G.** Nucleus targeted tdTomato. **H.** Membrane targeted tdTomato (red). **I.** Somato-dendritic targeted fusion of Kv2.1 and ChR2-EYFP. **J.** “Post-synaptic” targeted fusion of PSD95 and mNeonGreen. **K.** “Pre-synaptic” targeted fusion of synaptophysin and tdTomato. **L.** Mitochondria targeted dTomato. **M.** Imaging of recombinant rabies viruses expressing a “fluorescent timer” switching blue to red color over maturation time following infection. Scale bar: 10 µm.

**
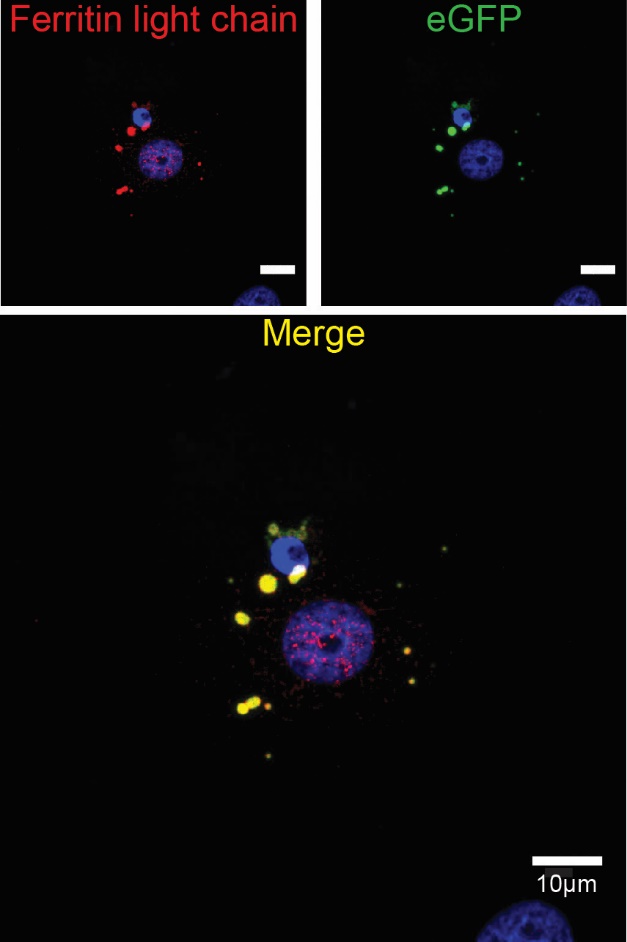
**

**Supplementary Figure 2: Recombinant rabies viruses express dual reporters.** Vero cells were infected with rabies virus expressing eGFP and ferritin light chain. At 48hpi cells were fixed and immunofluorescent assay targeting ferritin light chain was performed. Cells were imaged for eGFP (green fluorescent emission) and ferritin light chain (immunocytochemistry, red label). Nuclei were stained using DAPI.

**
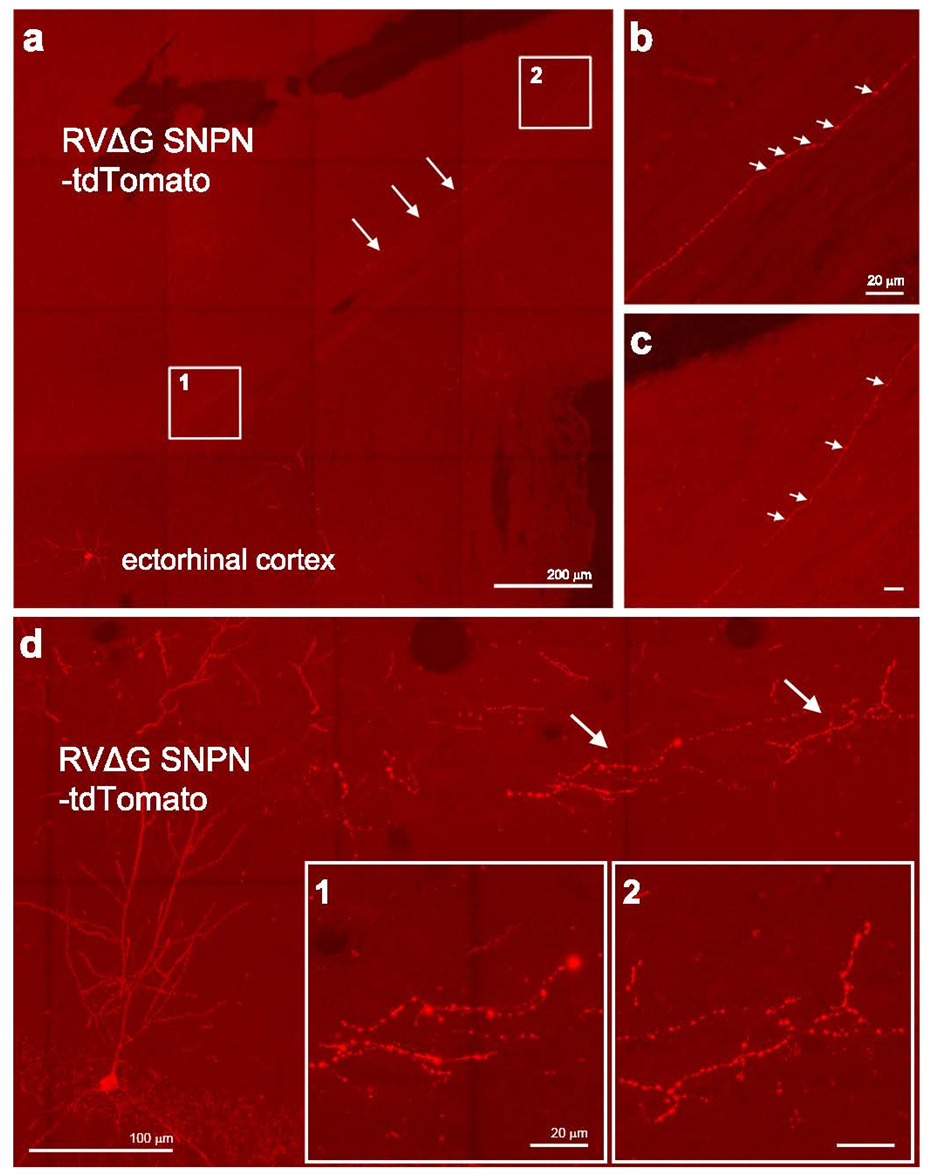
**

**Supplementary Figure 3. Recombinant rabies virus labels post-synaptic compartments with targeted fluorescent reporters. (a-c)** Long-range projecting axons and their boutons in ectorhinal cortex labeled by the RVΔG SNPN-tdTomato variant that is designed to target labels to pre-synaptic specializations by fusion to synaptophysin. Seven days following RVΔG SNPN-tdTomato injection into the subiculum, long-range projecting axons can be visualized in the ectorhinal cortex. The arrows in (a) indicate one segment of an axon. The boxes 1 and 2 in (a) are enlarged to show in (b) and (c), respectively. The arrowheads point to putative axonal boutons. (d) Axonal boutons labeled by the RVΔG SNPN-tdTomato variant in hippocampal CA1. The axon segments indicated by the arrows are shown in enlargement in the inserted boxes 1 and 2.


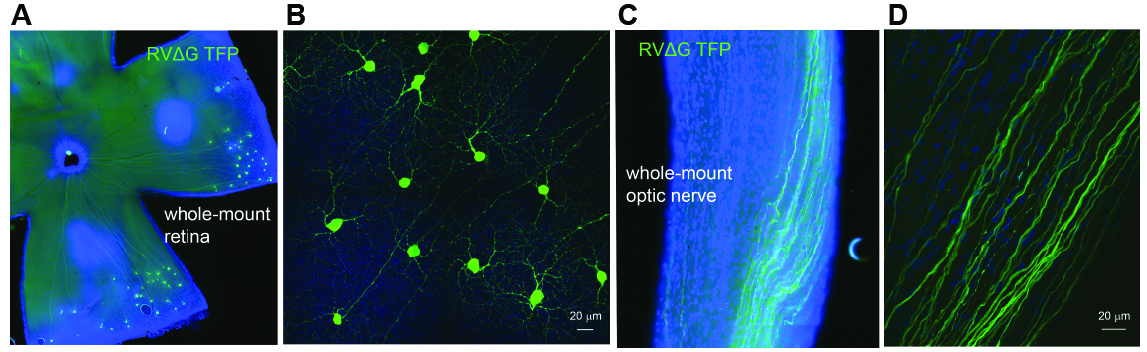


**Supplementary Figure 4. High level in vivo RVΔG-mediated fluorescent labeling of targeted neurons in the mouse retina.**

**(A-D).** RVΔG mTFP1 labeling of retinal ganglion cells and optical nerves following the RVΔG mTFP1 injection in mouse dorsal LGN.  A low power image of a whole-mount retina and peripheral retinal ganglion cells labeled with RVΔG mTFP1 is shown in **A**, and a high power confocal image of a portion of retinal ganglion cells is shown in (**B**).  Images of whole-mount optic nerves and axon bundles labeled with RVΔG mTFP1 are shown in (**C**) and (**D**), all sections are counterstained with DAPI (blue).

**
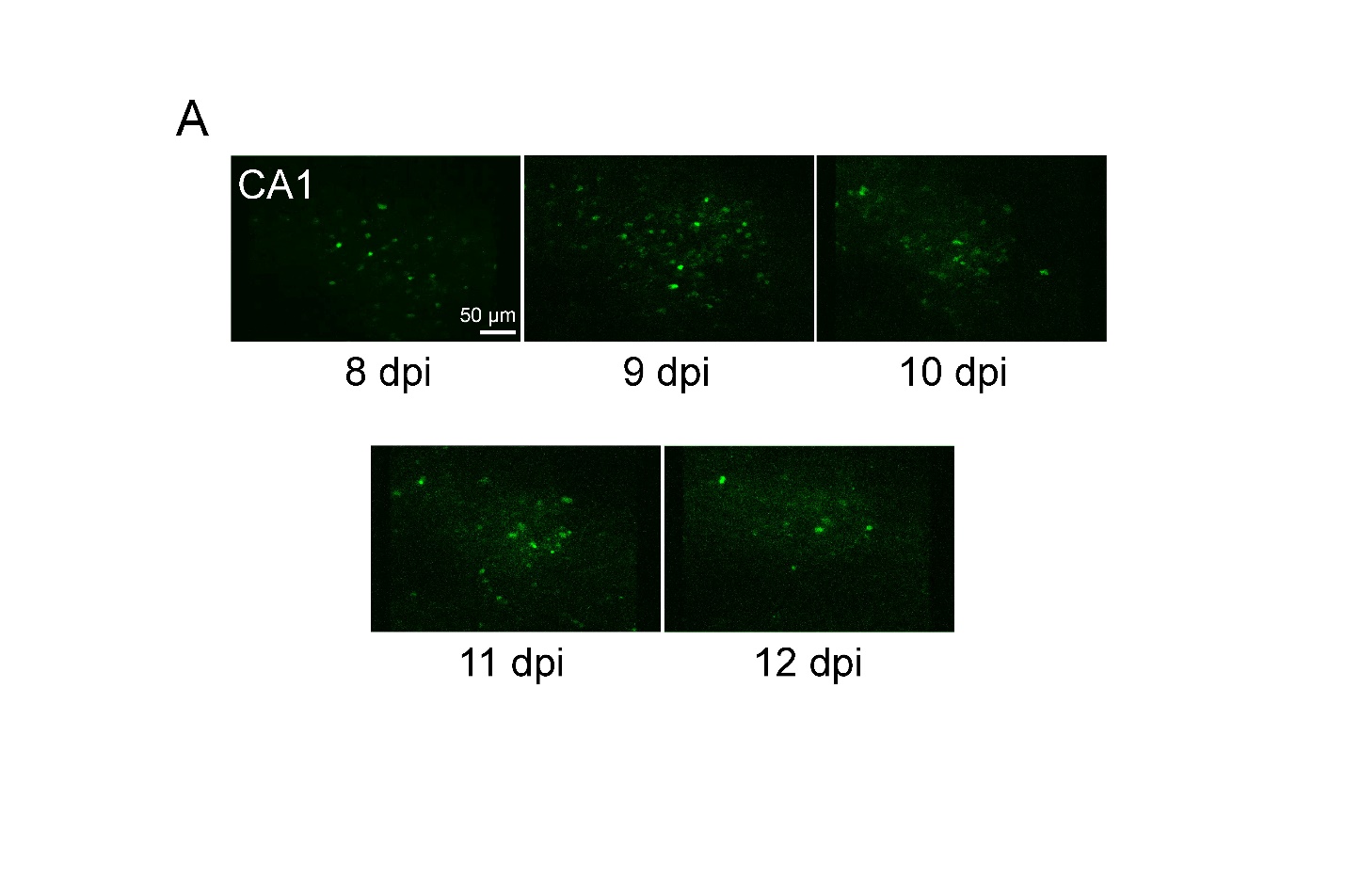
**

**Supplementary Figure 5. Enlarged views of RVΔG GCaMP7f infected CA1 neurons.**

Neurons in the same field of view show changes in morphology and GCaMP7f expression across 8-12 dpi. The scale bar represents 50 µm.

**Supplementary Movies**

**Supplementary Movies 1 and 2. Live imaging of mitochondria transport in cultured neurons using recombinant rabies viruses expressing targeted fluorescent reporters.**  Time-lapse imaging of motility of single/multiple mitochondria in axons of cultured superior cervical ganglionic neurons.

**Supplementary Movie 3.** **Two-photon recording of CA1 neurons infected with RVΔG GCaMP7f in mouse #09 on day 9 after the rabies viral injection (9 dpi).** The scale bar represents 50 μm. The playback speed is 8x.

**Supplementary Movie 4.** **Correlated light microscopy (LM), x-ray microscopy (XRM) and serial block-face scanning EM (SBEM) of RVΔG-mediated, emGFP-ferritin neuronal labeling**.
